# Effector gene birth in plant parasitic nematodes: Neofunctionalization of a housekeeping glutathione synthetase gene

**DOI:** 10.1371/journal.pgen.1007310

**Published:** 2018-04-11

**Authors:** Catherine J. Lilley, Abbas Maqbool, Duqing Wu, Hazijah B. Yusup, Laura M. Jones, Paul R. J. Birch, Mark J. Banfield, Peter E. Urwin, Sebastian Eves-van den Akker

**Affiliations:** 1 Centre for Plant Sciences, Faculty of Biological Sciences, University of Leeds, Leeds, United Kingdom; 2 Dept. of Biological Chemistry, John Innes Centre, Norwich Research Park, Norwich, United Kingdom; 3 Cell and Molecular Sciences Group, Dundee Effector Consortium, James Hutton Institute, Invergowrie, Dundee, United Kingdom; 4 School of Life Sciences, University of Dundee, Dundee, United Kingdom; University of California Davis, UNITED STATES

## Abstract

Plant pathogens and parasites are a major threat to global food security. Plant parasitism has arisen four times independently within the phylum Nematoda, resulting in at least one parasite of every major food crop in the world. Some species within the most economically important order (Tylenchida) secrete proteins termed effectors into their host during infection to re-programme host development and immunity. The precise detail of how nematodes evolve new effectors is not clear. Here we reconstruct the evolutionary history of a novel effector gene family. We show that during the evolution of plant parasitism in the Tylenchida, the housekeeping glutathione synthetase (GS) gene was extensively replicated. New GS paralogues acquired multiple dorsal gland promoter elements, altered spatial expression to the secretory dorsal gland, altered temporal expression to primarily parasitic stages, and gained a signal peptide for secretion. The gene products are delivered into the host plant cell during infection, giving rise to “GS-like effectors”. Remarkably, by solving the structure of GS-like effectors we show that during this process they have also diversified in biochemical activity, and likely represent the founding members of a novel class of GS-like enzyme. Our results demonstrate the re-purposing of an endogenous housekeeping gene to form a family of effectors with modified functions. We anticipate that our discovery will be a blueprint to understand the evolution of other plant-parasitic nematode effectors, and the foundation to uncover a novel enzymatic function.

## Author summary

Plants and their pathogens/parasites are locked in an evolutionary arms race, with considerable attention directed towards the specific functions of the parasites’ “weapons”: the effectors. While we are beginning to understand these functions, we have very little understanding of how plant parasitic nematodes have bolstered their effector repertoire. Here we provide an example of how plant parasites of global economic importance have populated their effector repertoire by the unprecedented duplication and subsequent re-deployment of the endogenous housekeeping gene, glutathione synthetase. We hypothesise that many aspects of the “weaponization” programme detailed here will be common to the genesis of other plant-parasitic nematode effectors. Given that parasitic nematodes deploy a battery of effectors, many arising from the adaptation of either endogenous genes or loci acquired by horizontal gene transfer, this paradigm will have substantial impact on the effort to understand and ultimately undermine devastating USDA and EPPO quarantine organisms.

## Introduction

The ability of nematodes to exploit living plants as a food resource has arisen independently in four of the twelve major lineages of the phylum Nematoda [1]. As a result, plant-parasitic nematodes occupy a diverse range of niches and climates, and infect a wide range of host species globally. Clade 12 of the phylum encompasses representatives of all major modes of parasitism; migratory ectoparasites, migratory endoparasites, and the most economically important and highly specialized obligate biotrophs—the sedentary endoparasites [2]. The latter induce the re-differentiation of root cells to form a unique nutrient-rich feeding site which is maintained for several weeks in a prolonged biotrophic interaction. For the cyst and reniform nematodes this takes the form of a large syncytium that arises through local cell wall dissolution and fusion of neighbouring protoplasts. Nematodes deploy hundreds of effector proteins to induce profound molecular and physiological changes associated with feeding site induction and maintenance. The majority of all described effectors are secreted from three pharyngeal gland cells (one dorsal and two subventral) through a hollow, protrusible needle-like stylet, into the plant.

While the basis for the evolution of nematode parasitism is largely unresolved and widely debated [3, 4], it is likely that a series of evolutionary transitions gave rise to the biologically complex sedentary plant endoparasites [5, 6]. Surprisingly little is known about the genetic changes that occurred with these transitions. In general, parasites lose functions as they further rely on their host. For the sedentary endoparasitic cyst nematodes, this is evidenced by a reduction in genes involved in detoxification of xenobiotic compounds, and the absence of whole classes of antibacterial and antifungal genes [7]. However, concurrent with this process, cyst nematodes have evolved a large repertoire of effectors that facilitate their remarkable abilities to suppress plant immunity and induce plant cells to re-differentiate into a novel tissue. The evolution of sedentary endoparasitism must therefore be additionally characterized by acquisition of novel functions.

Both acquisition of new genes by horizontal transfer [8] and selective expansion of particular gene families [9] are associated with the evolution of a parasitic lifestyle. For example, a large expansion of the astacin protease and CAP gene families coincided with the emergence of parasitism in the animal parasitic *Strongyloides* nematodes [10] whilst gene duplication is proposed to have been an important driver of parasitism in the Orobanchaceae plant parasites [11]. Examples of such expansions are also present in the genome of the potato cyst nematode *Globodera pallida*. The most unusual expansion comprises more than 50 predicted genes with similarity to glutathione synthetase enzymes [12]: almost all eukaryotes possess only a single glutathione synthetase gene.

Glutathione, the tri-peptide γ-L-glutamyl-cysteinyl-glycine, is the main low molecular weight thiol antioxidant in both plant and animal cells, often present at millimolar concentrations *in vivo* [13]. It is synthesised in a two-step, ATP-dependent, process: glutamate-cysteine ligase (GCL) catalyses the formation of γ-glutamylcysteine (γ-EC) from glutamate and cysteine, followed by the addition of glycine by glutathione synthetase (GS) to form glutathione. Glutathione has a fundamental, multifunctional role in modulating the redox status of cells, protecting them against oxidants and electrophiles, and detoxifying xenobiotics [14]. In both plants and animals, glutathione has a role in cellular defence responses against abiotic and biotic stress [15, 16]. Glutathione has been particularly implicated in plant responses to pathogens. Its abundance decreases during compatible interactions but it accumulates in response to avirulent pathogens [17] and can induce the expression of plant defence genes [18, 19]. Glutathione is also involved in biotic stress signalling [20], in particular NPR1-dependent/independent salicylic acid (SA)-mediated pathways [21, 22] and plants with reduced glutathione levels are generally more susceptible to pathogens and herbivorous insects [23, 24].

Given the diverse functions of glutathione it is clear that an expanded family of glutathione synthetases could be involved in numerous aspects of the plant-nematode interaction. Here we demonstrate that the expansion is not restricted to *G*. *pallida* and multiple subsequent expansions of GS genes have co-occurred with the evolution of complexity in plant-nematode interactions. The most recent expansion has been coupled with a diversification of structure and biochemical function that has given rise to enzymes that are introduced into the host cell, and likely possess novel substrate specificities.

## Results

### Two successive glutathione synthetase gene family expansions have occurred during the evolution of cyst and reniform nematode obligate biotrophic interactions

**Fig 1:**
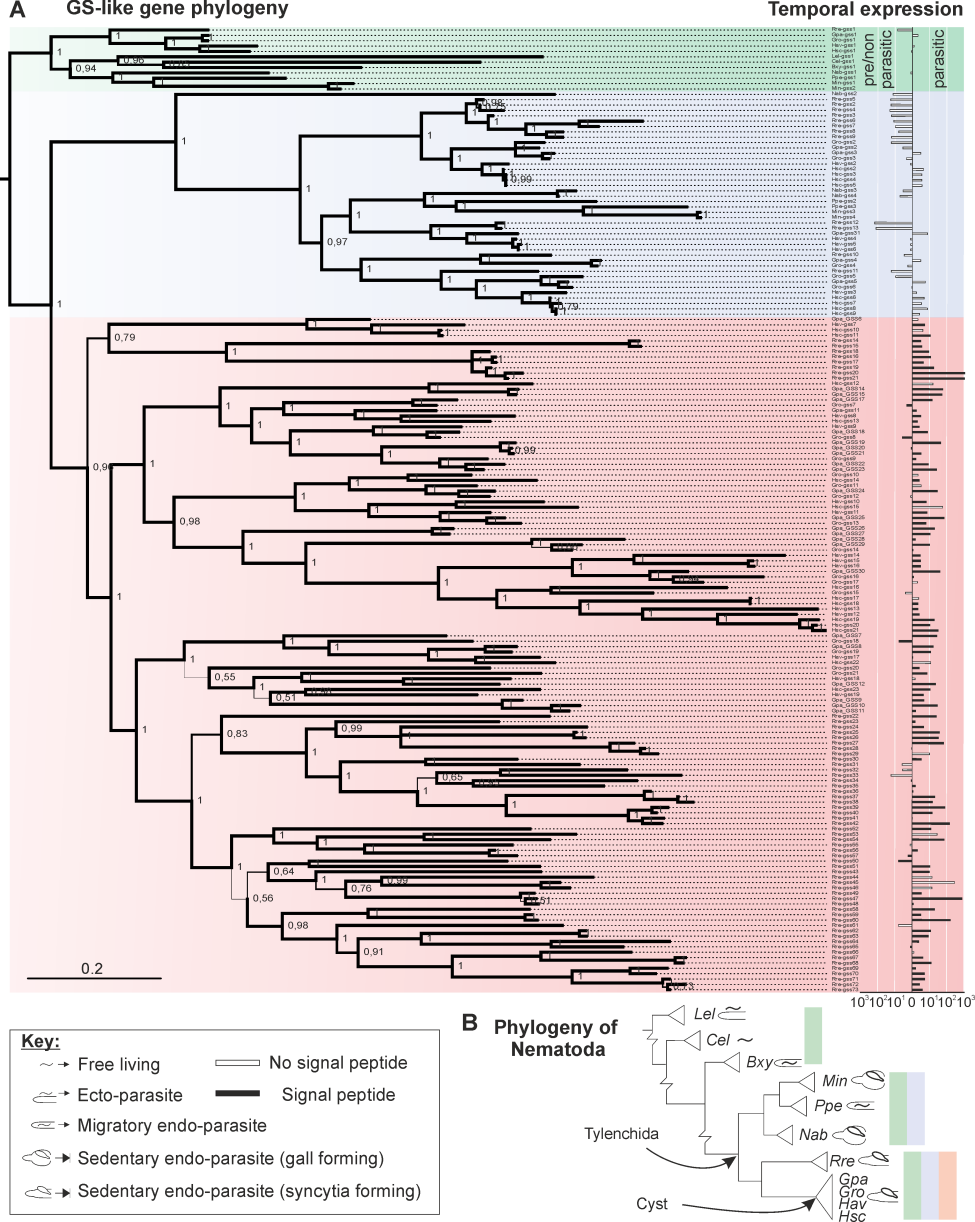
Two successive glutathione synthetase gene family expansions have occurred during the evolution of cyst and reniform nematode obligate biotrophic interactions. **A**) A representation of a Bayesian phylogenetic tree based on a protein alignment of 180 glutathione synthetase (GS) like genes from eleven nematode species. The phylogeny is divided into three well supported Clades (node labels indicate support values for 2.5 million iterations, branch line width is scaled by support). Clade 1 (green) contains at least one sequence from each nematode species analysed. Clade 2 (blue) contains the first expansion of GS genes and exclusively comprises sequences from nematode species which spend the majority of their life within their plant host (and includes both migratory and sedentary endo-parasites). Clade 3 (red) contains the second, more recent and more diverse expansion and comprises cyst and reniform nematode genes only. Expression data for *G*. *pallida*, *G*. *rostochiensis*, *R*. *reniformis*, *N*. *aberrans*, *H*. *avenae*, and *H*. *schachtii* is shown adjacent to each sequence, as the fold-change between pre/non-parasitic and parasitic stages. See S2 Fig for a more detailed analysis of *G*. *pallida* GS-like gene expression across the life cycle. Black bars indicate those sequences that encode a canonical signal peptide for secretion. **B)** Schematic phylogeny of the phylum Nematoda [26, 72] with a key depicting the different modes of parasitism, and the species analysed: *Lel–Longidorus elongatus*; *Cel*–*Caenorhabditis elegans*; *Bxy–Bursaphelenchus xylophilus; Min*–*Meloidogyne incognita*; *Ppe*–*Pratylenchus penetrans*; *Nab*–*Nacobbus aberrans*; *Rre*–*Rotylenchulus reniformis*; *Gpa*–*Globodera pallida*; *Gro*–*Globodera rostochiensis*; *Hav*–*Heterodera avenae; Hsc*–*Heterodera schachtii*. Coloured bars indicate the GS Clades that those species contribute sequences to.

We analysed the genomes and/or transcriptomes of eleven plant-parasitic and non-plant-parasitic nematode species (*Caenorhabditis elegans*, *Bursaphelenchus xylophilus*, *Longidorus elongatus*, *Pratylenchus penetrans*, *Meloidogyne incognita*, *Nacobbus aberrans*, *Rotylenchulus reniformis*, *Globodera rostochiensis*, *G*. *pallida*, *Heterodera schachtii* and *H*. *avenae)* to explore the expansion of genes encoding glutathione synthetase-like domains (Pfams PF03917 and PF03199 –Fig 1). Although almost all plant and animal genomes encode only a single housekeeping glutathione synthetase gene (GS), some plant-parasitic nematode genomes encode an unprecedented number (up to ~70 in *R*. *reniformis*). A Bayesian phylogeny inferred from an alignment of all 180 GS-like loci from these eleven species reveals two major expansion events, resulting in three Clades (Fig 1; complete GS-like gene sequences are available under Dryad accession doi:10.5061/dryad.7vd0160). Clade 1 contains one sequence from each nematode in the phylogeny (with the exception of *M*. *incognita* that, due to its polyploid genome, contributes two genes to the Clade [25]). It includes the only sequence from the free living nematode *C*. *elegans*, and the relative topology of the sequences in Clade 1 is similar to the most recent single and multi-gene phylogenies of plant parasitic nematodes for those species included [1, 26]. Taken together, this suggests that Clade 1 contains the housekeeping progenitor GS of each species.

The first expansion of GS-like genes (Clade 2) was present in the last common ancestor of present day migratory and sedentary plant endoparasites belonging to the order Tylenchida. Species vary in the number of Clade 2 genes from two (*P*. *penetrans* and *M*. *incognita*) to 12 (*R*. *reniformis*), with a mean, mode, and median of ~5. Clade 2 does not include sequences from the free living nematode *C*. *elegans*, the migratory ectoparasite *L*. *elongatus* or the non-Tylenchid migratory endoparasite *B*. *xylophilus*. We note that many Clade 2 GS share a short and somewhat variable C-terminal extension of the approximate consensus sequence P[A|S][A|S][E|Q][F|L], which has no known function (S1 Fig). This peptide is absent in all other clades and is not recognised as a canonical signal by TargetP.

A second larger expansion of GS-like genes (Clade 3) was present in the last common ancestor of the syncytia-forming cyst and reniform nematodes, which both lie on one side of a major bifurcation in the evolution of plant-sedentary endoparasitism (Fig 1). On average, sequences in Clade 3 share 36% protein identity with one another, and are much more diverse than those of Clades 1 and 2 (51% and 52% identity respectively) despite encompassing a narrower range of species. The fact that 68% of *G*. *pallida* and *G*. *rostochiensis* Clade 3 GS-like genes (26/38) are more similar to a Clade 3 GS from another cyst nematode in the phylogeny than they are to another Clade 3 GS from their own species, suggests that the majority of the diversity in cyst nematode Clade 3 GS was probably present in their last common ancestor. The *R*. *reniformis*, *H*. *avenae*, and *H*. *schachtii* GS-like gene complements were largely or entirely assembled from *de novo* transcriptome data rather than genomic data, precluding similar conclusions. Nevertheless we note that 8/14 *H*. *avenae* Clade 3 GS are more similar to a Clade 3 GS from other cyst nematodes than they are to other Clade 3 GS from their own species. In contrast all *R*. *reniformis* Clade 3 GS are contained within two sub-clades, and are more similar to other *R*. *reniformis* Clade 3 GS than Clade 3 GS from the cyst nematodes.

Given that species which have GS-like genes from the first expansion (Clade 2) have retained the progenitor housekeeping gene (Clade 1), and those that have GS-like genes from the second expansion (Clade 3) have retained both Clade 1 and 2, this suggests that the role of each expansion does not supersede its predecessor(s). They likely represent gain of novel function during the evolution of endoparasitism.

### New GS-like paralogues are redeployed during parasitism as effectors

Each expansion of GS-like genes has a distinctive temporal expression pattern compared to its predecessor (Fig 1A). Clade 2 GS-like genes exhibit up-regulation predominantly in pre/non-parasitic stages, while Clade 3 GS-like genes are highly up-regulated, and often specifically expressed, during the plant-parasitic stages of endoparasitism. Given that the average within-species identity of *G*. *pallida* GS-like coding sequences is 49%, we can be confident in the distinct expression patterns of each Clade by RNAseq mapping. The disparity in differential temporal expression of GS-like genes further supports their functional diversification at different points of the life cycle. More detailed transcriptional analysis of all *G*. *pallida* GS-like genes throughout the lifecycle demonstrates that those of Clade 3 are highly up-regulated upon early interaction with the host plant and this expression is generally maintained throughout the parasitic stage (7, 14, 21, 28, and 35 days post infection; S2 Fig). This is strongly indicative that the most recently evolved Clade 3 GS-like paralogues are involved in prolonged sedentary endoparasitism.

Analysing the promoters of *G*. *pallida* and *G*. *rostochiensis* GS-like genes, we discovered that specifically those in Clade 3 harbour multiple copies of the DOG box, a promoter motif associated with dorsal gland cell expression in *Globodera* spp. [27, 28]. *Globodera* Clade 1, 2 and 3 GS-like gene promoters contain on average 0.5, 0.2, and 1.31 DOG boxes respectively (S1 Table). While there is no correlation between the number of DOG box motifs per promoter and temporal expression of the corresponding gene (R^2^ = 0.03 and [28]), Clade 3 GS-like genes with >1 DOG box in the first 1000 bp upstream of the coding start site are approximately twice as highly up-regulated during parasitism as those in Clade 3 without DOG boxes (24-fold (n = 16), 13-fold (n = 6)).

**Fig 2:**
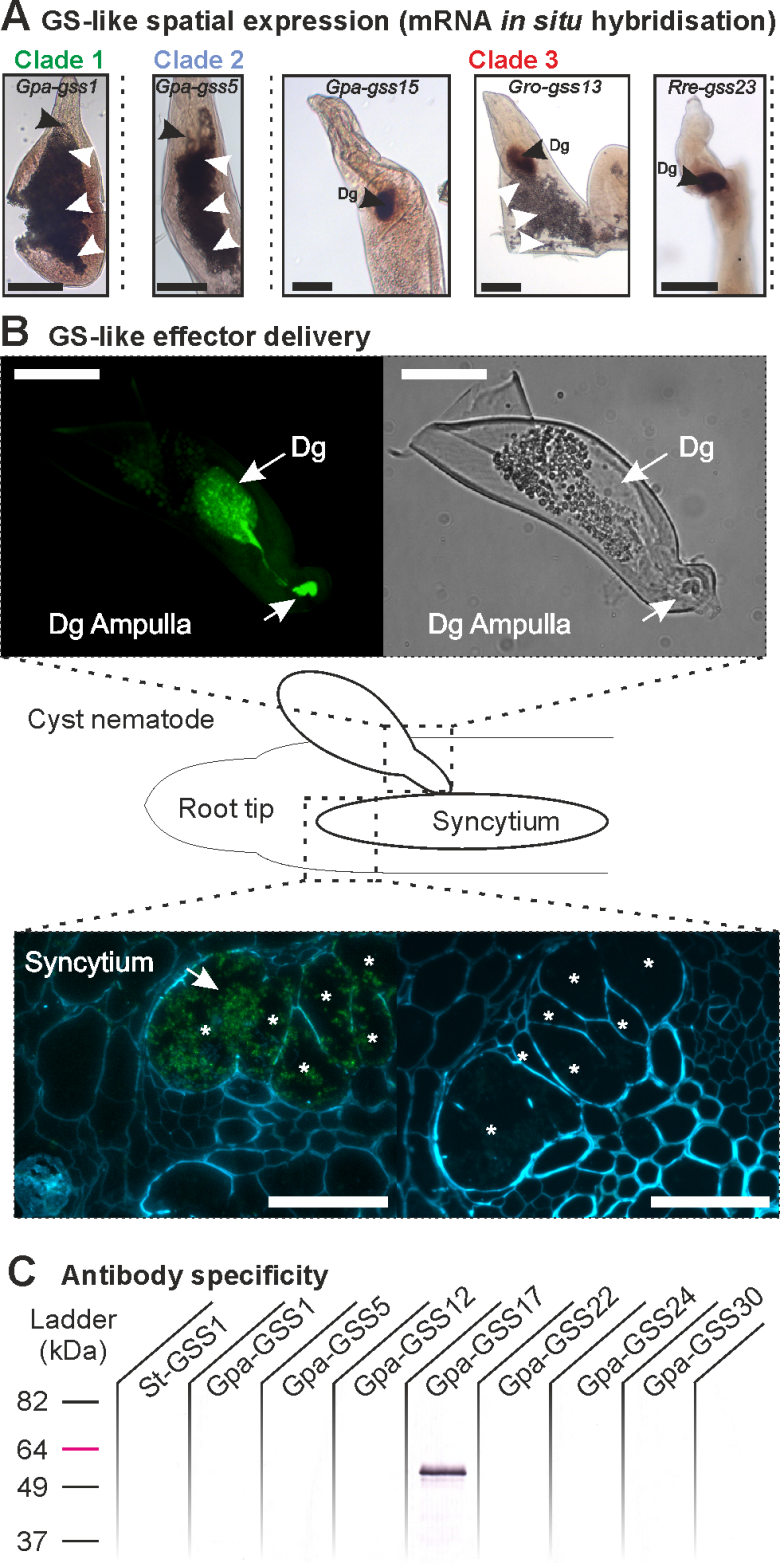
New GS-like paralogues are redeployed during parasitism as effectors. **A**) Examples of spatial expression patterns (*in situ* hybridisation) of GS-like gene members of Clades 1, 2, and 3 (See S3 Fig for additional examples and negative controls). *Globodera pallida* Clade 1 and 2 GS genes are expressed in the intestine (white arrowheads). Clade 3 GS-like genes are specifically expressed in the dorsal gland cell (Dg) of *G*. *pallida*, *G*. *rostochiensis* and *R*. *reniformis* (left to right). Scale bars = 100 μm. **B**) An affinity-purified antibody raised against *G*. *pallida* Clade 3 GS-like effector Gpa-GSS17 recombinant protein was used for immunolocalisation (green fluorescence). Gpa-GSS17 is localised in the dorsal gland cell (Dg) of parasitic nematodes, the cytoplasmic gland extension and the ampulla at the base of the stylet where secretions accumulate prior to their release (top) Scale bars = 50 μm. The same native protein was localised within the syncytial feeding site (asterisks) induced by the nematode in a potato root (green fluorescence, bottom left). No such localisation was seen with the 2^o^ antibody control (bottom right). Cell walls are stained blue. **C**) Western blot to determine antibody specificity. The anti-Gpa-GSS17 antibody specifically recognises Gpa-GSS17, and does not recognise other Clade 3 GS-like proteins tested, other plant-parasitic nematode GS proteins tested (Clades 1 and 2), nor the endogenous potato GS (St-GSS1).

The presence of the DOG box in the promoter of previously uncharacterised genes is used to predict dorsal gland cell-expressed effectors in cyst nematodes [27]. Consistent with DOG box enrichment, all GS-like genes from Clade 3 analysed by *in situ* hybridisation were highly and specifically expressed in the secretory dorsal gland cells in a range of parasitic stage nematodes (Fig 2A and S3 Fig), while Clade 1 and Clade 2 GS-like genes were expressed in both female and male nematodes, with often preferential expression in the intestine (Fig 2A and S3 Fig. Gland cell expression holds true for both major groups of nematodes that encode Clade 3 GS, the cyst and the reniform nematodes (Fig 2A). The gland cells are the major effector-producing tissue, and secreted proteins expressed in the dorsal gland cell are delivered into the host plant [29, 30]. Strikingly, almost all Clade 3 GS-like genes, but none of those from Clades 1 and 2, encode a canonical N-terminal secretion signal (Fig 1A, black bars). These 102 putatively secreted Clade 3 GS-like proteins are the only known GS from the plant or animal kingdoms that possess such a signal. While we cannot rule out the possibility that Clade 3 GS proteins that do not encode a signal peptide are in fact secreted by non-canonical pathways, as presumed for some nematode effectors (eg. [20, 31]), we restrict our analysis in Clade 3 to signal peptide-encoding GS-like genes. Taken together, a clear distinction can be made between Clade 3 GS-like genes and those of Clades 1 and 2 based on their promoters, their spatial expression patterns, the presence of a signal peptide, and the likelihood of the sequence being upregulated in parasitic stages.

Tissue-specific expression of a putatively secreted protein in secretory dorsal gland cells is a strong indicator of a nematode effector that is delivered *in planta*. To confirm this, an affinity-purified polyclonal antibody was raised against recombinant Gpa-GSS17 and shown to be specific for this protein (Fig 2C). Using this antibody we are able to detect the abundant presence of Gpa-GSS17 in the large dorsal gland cell of parasitic stage *G*. *pallida* nematodes, the cytoplasmic gland extension, and the ampulla at the base of the stylet where secretions accumulate prior to their release (Fig 2B). The same native protein was delivered into the host across the plasma membrane, and is localized within the syncytial feeding site induced by the nematode in potato roots (Fig 2B). No similar fluorescence was observed with the FITC-labelled 2^o^ antibody control. Hereafter, Clade 3 genes are thus referred to as GS-like effector genes. While it is highly unusual for a 100 kilodalton pathogen effector to be translocated to the host cell cytoplasm (GS-like effectors are obligate homo-dimers of 50 kDa per subunit, S4 Fig), plant parasitic nematodes clearly have the ability to construct organelle-sized structures inside the host cell at the plasma membrane where it meets the stylet orifice (reviewed in [32]). The demonstrations that a native GS-like effector protein is secreted from the nematode, delivered into the host, translocated across the host plasma membrane, and localised within the host cell during parasitism are strong evidence of involvement in parasitism.

Taken together, this illustrates how these plant-parasitic nematodes have exploited multiple gene gain events to deploy a novel effector family during parasitism. Interestingly, this programme of effector evolution is potentially more broadly applicable to other well-documented gene gain events in plant-parasitic nematodes: horizontally transferred genes must acquire a similar set of genetic attributes in order to be deployed as effectors during parasitism [33].

### GS-like genes are not just re-deployed, but are re-purposed

**Fig 3:**
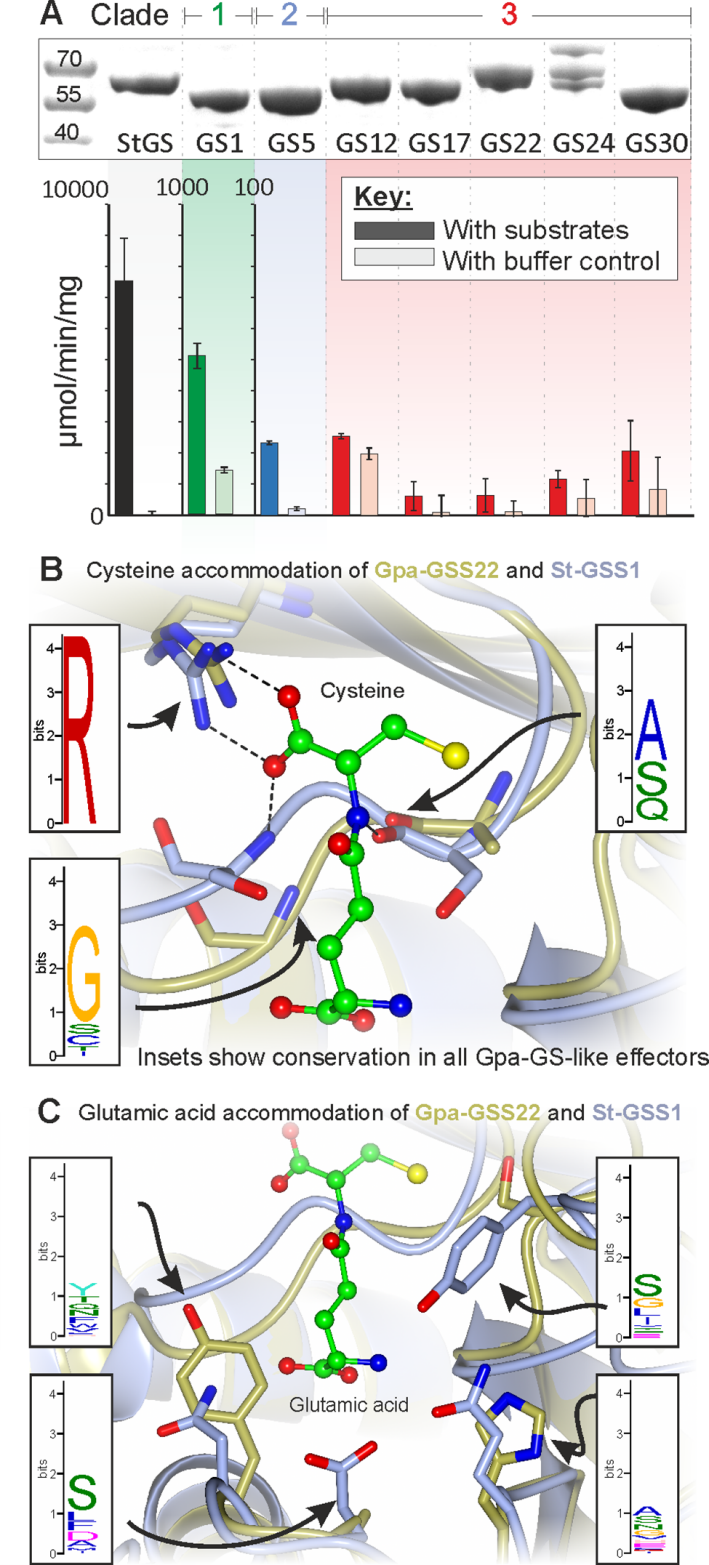
Cyst nematode GS-like genes are re-purposed to carry out a novel function. **A)** Purified protein for St-GSS1, Gpa-GSS1, 5, 12, 17, 22, 24 and 30 were tested for glutathione synthetase activity by measuring phosphate release from ATP in the presence of canonical substrates (γ-EC, glycine and ATP). To determine specific activity, rates are presented with subtraction of buffer controls in S2 Table. **B)** and **C)** Comparison of residues in the di-peptide binding pocket of Gpa-GSS22-closed and St-GSS-closed, with inset the variation at these positions in all other *G*. *pallida* Clade 3 GS-like effectors. **B)** In St-GSS1, the cysteine of the di-peptide substrate (γ-EC) is coordinated by the side chain of an arginine (top left), and the backbone of two serines (bottom left and bottom right). The arginine is conserved in Gpa-GSS22 and all GS-like effectors (inset). The two serines are not conserved in sequence in either Gpa-GSS22 or the remainder of Clade 3 (inset), but the equivalent residues are preferentially small and uncharged amino acids that do not vary greatly in the remainder of Clade 3 (inset). **C)** In St-GSS1, the glutamic acid of γ-EC is coordinated exclusively by side chain interactions with a number of charged residues. All of these residues are different in Gpa-GSS22, and these positions are highly variable across Clade 3 (inset).

To explore the catalytic capacity of nematode GS-like genes, the *G*. *pallida* representative of Clade 1 (*Gpa-gss1*), one representative from Clade 2 (*Gpa-gss5*) and a number from Clade 3 (*Gpa-gss12*, *Gpa-gss17*, *Gpa-gss22*, *Gpa-gss24 and Gpa-gss30*) were heterologously expressed in, and their proteins purified from, bacteria (Fig 3A). For comparison, the only GS gene in the corresponding plant host of *G*. *pallida* (*Solanum tuberosum*, *St-gss1*), was similarly expressed and its product purified. All GS-like proteins were purified as obligate homo-dimeric pairs (S4 Fig). Purified GS were tested for canonical glutathione (Glu-Cys-Gly) synthetic capacity using a spectrophotometric assay where phosphate release from ATP is used as a molar equivalent proxy for glutathione synthesis from the substrates γ-glutamyl-cysteine (γ-EC) and glycine. Using this approach, the maximum rate of the host GS (St-GSS1) phosphate release was 7532 (±1358) umol/mg/min (S2 Table), consistent with a previous report for Arabidopsis GS of ~7500 umol/mg/min [34], demonstrating the suitability of the assay.

Remarkably, each stage of the evolutionary process that gave rise to GS-like effectors has witnessed at least a 10-fold reduction in apparent glutathione synthetic rate, such that Clade 3 GS-like proteins have not retained canonical enzyme activity (Fig 3 and S2 Table). We argue this loss of canonical activity is probably associated with a gain of non-canonical activity. We have analysed 180 GS-like protein sequences, each approximately 500 amino acids in length, from 11 species across the phylum. Within the context of an average sequence identity of only ~34%, there are just 5 residues that are absolutely conserved in all 180 sequences: three of these are in the ATP binding pocket, and one of these is required for catalysis. Perfect conservation of the catalytic residue in itself implies individual catalytic functionality. Given that GS effectors have no appreciable rate of ATP turnover when supplied with canonical substrates, yet all display perfect conservation at the catalytic arginine, we infer that GS-like effectors possess a distinct catalytic activity, which may involve alternative substrate(s).

GS-like enzymes that vary in substrate usage have been described in plants, yet in all cases this variation is restricted to the terminal amino acid: the same γ-EC backbone is universally used as a scaffold [35, 36]. For example, the homo-glutathione synthetase (hGS) of *Glycine max* (soya bean) catalyses the addition of β-alanine to γ-EC giving rise to homoglutathione (hGSH, γ-glu-cys-β-ala). The ability of purified GS-like proteins to incorporate a range of natural and non-natural terminal amino acids onto the γ-EC backbone was tested. St-GSS1, Clade 1 Gpa-GSS1, and Clade 2 Gpa-GSS5 all exhibited a strong preference for glycine, and a high affinity for γ-EC (S5 Fig). In contrast, none of the Clade 3 GS-like effectors accepted any of the tested terminal amino acid substrates in combination with the γ-EC backbone. One possible explanation for this is that Clade 3 GS-like effectors represent a novel diversification at the site of the di-peptide acceptor.

**Fig 4:**
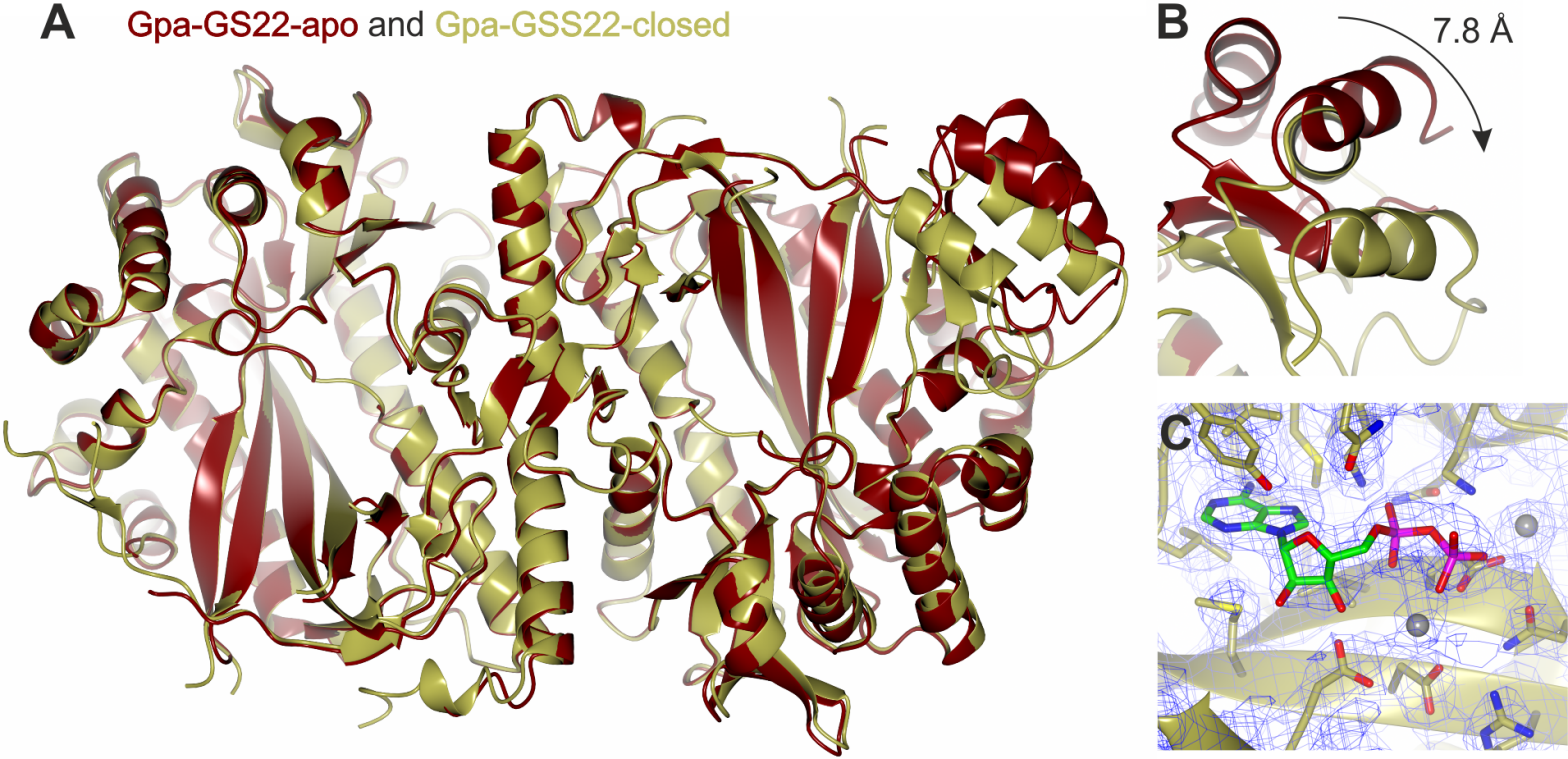
The crystal structure of *Globodera pallida* GS-like effector Gpa-GSS22. **A)** The crystal structure of Gpa-GSS22 is composed of a homodimeric molecule in both its open (apo (red)) and ADP-bound closed (gold) conformations. **B)** The two helix bundle that constitutes the ATP grasp fold undergoes a 7.8 Å conformational change on binding ADP to close over the active site. **C)** The presence of electron density (blue mesh) in the active site is consistent with a single ADP molecule and two magnesium ions per subunit.

To create a structural basis for the exploration of novel substrate specificities, we initially solved the first crystal structure of a canonical plant GS (St-GSS1, *S*. *tuberosum*, host of *G*. *pallida*) in complex with ADP and the canonical di-peptide substrate γ-EC (2.5 Å, PDB 5OES). The crystal structures of two non-canonical parasite GS-like effectors were subsequently solved: Gpa-GSS30 in its apoform (2.6 Å, PDB 5OET) and Gpa-GSS22 in both its apoform (2.2 Å, PDB 5OEV) and ADP-bound closed conformation (2.6 Å, PDB 5OEU, S3 Table). Comparison between Gpa-GSS22-open and–closed reveals a functioning ATP grasp fold (Fig 4).

**Fig 5:**
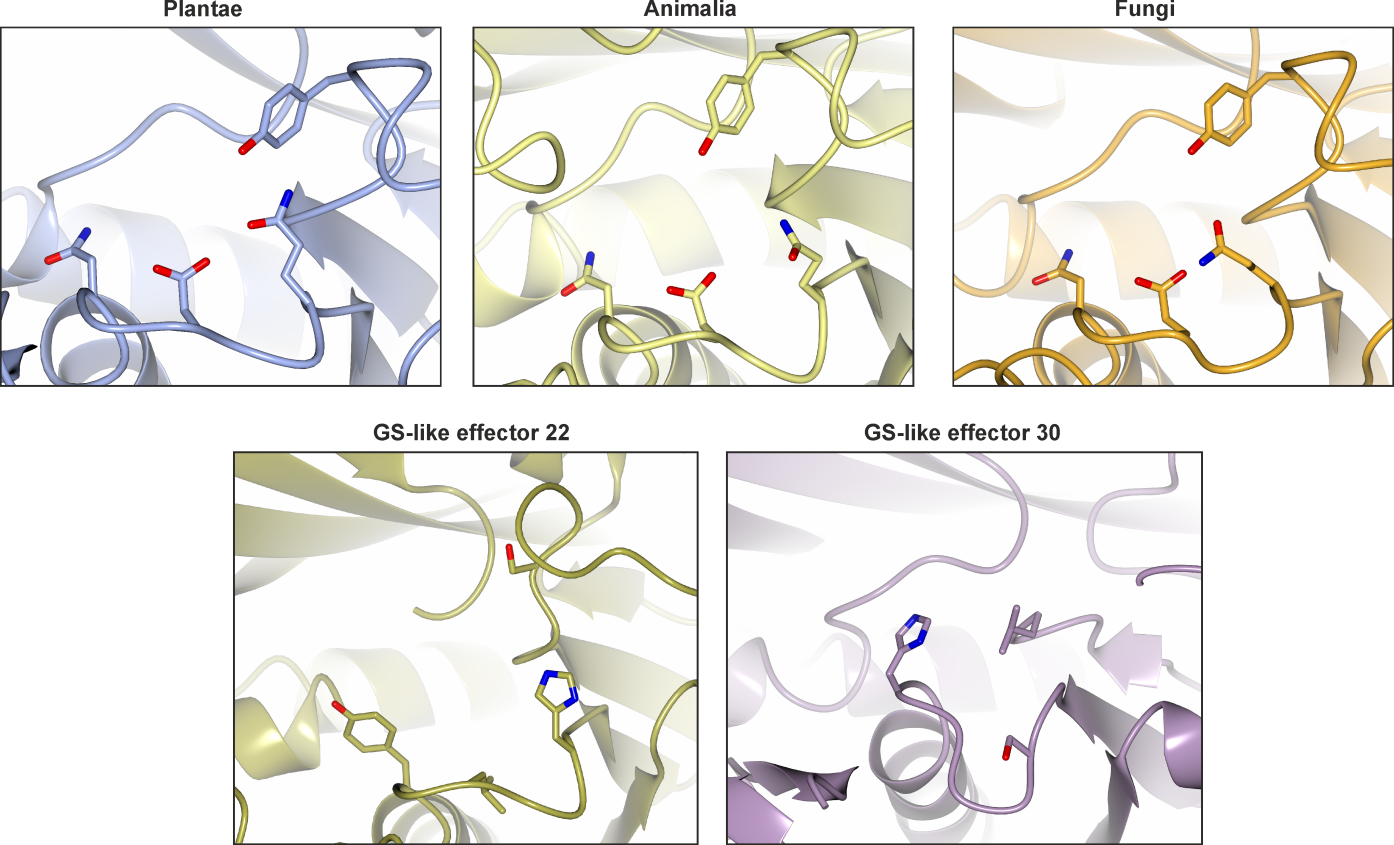
Comparison of residues in the glutamate binding pocket of canonical GS with the same positions in nematode GS-like effectors. The canonical arrangement in St-GSS1 surrounding the glutamic acid of γ-EC has been conserved for ~1 billion years of evolution in three kingdoms: Plantae (*Solanum tuberosum* St-GSS1-closed, PDB 5OES), Fungi (*Saccharomyces cerevisiae*, PDB 1M0T), and Animalia (*Homo sapiens* GSS1, PDB 2HGS). Both solved structures of GS-like effectors (Gpa-GSS22 and Gpa-GSS30) show a non-canonical arrangement that is highly unusual among Eukaryotes. Together with the conservation in the remainder of the di-peptide substrate binding pocket, and the conservation in the entire ATP binding pocket, this diversification likely indicates novel substrate usage.

Residues in the ATP binding pocket of St-GSS1 are highly conserved in sequence and position in the Gpa-GSS22 structure (13/15 residues, S4 Table), and similarly conserved in sequence across *G*. *pallida* Clade 3 (structure guided alignment, ~12/15, n = 24, S4 Table). In contrast, there is considerably more variation in the di-peptide binding pocket of *G*. *pallida* GS-like effectors, yet, this variation is not evenly distributed around the pocket. The position of γ-EC in St-GSS1 is coordinated at both the glutamate and the cysteine residue. Two of the three residues that coordinate the cysteine interact with the C-alpha backbone. Corresponding residues in all *G*. *pallida* GS-like effectors are not conserved in sequence but are preferentially small and uncharged (Fig 3B inset), while the third residue, arginine, is 100% conserved (Fig 3B and S4 Table): consistent with permitting interactions with a cysteine residue. However, the glutamate of the di-peptide is exclusively coordinated by interactions with charged side chains of residues in the binding pocket (R, E, 2xQ, N, and Y). All six of these positions are substituted in Gpa-GSS22 and Gpa-GSS30, thus suggesting that the lack of canonical activity is because γ-EC is not accepted despite the potential for cysteine to be accommodated (Fig 3C and S4 Table). Variability in the glutamate portion of the di-peptide binding pocket is ubiquitous in *G*. *pallida* Clade 3 GS: of the 24 GS-like effectors there are 21 different amino acid compositions in these 6 positions, not one of which is canonical (S4 Table). Such diversity is highly unusual among Eukaryotes: a canonical arrangement has been conserved in GS enzymes for ~1 billion years of evolution in three kingdoms of life (Fig 5, Plantae (St-GSS1-closed, PDB 5OES), Fungi (*Saccharomyces cerevisiae*, PDB 1M0T), and Animalia (*Homo sapiens* GSS1, PDB 2HGS)).

In summary, conserved amino acids are only absent from one half of the acceptor di-peptide binding pocket. The space that would accommodate the cysteine thiol—the “active residue” of glutathione—is well conserved in sequence and structure, and is non-variable in all GS-like effectors tested. Coupled with the high degree of conservation in the ATP-binding pocket, the functioning ATP-grasp fold, and the perfect conservation of a catalytic residue, these data support the hypothesis that the loss of canonical glutathione synthetic activity is associated with a gain of non-canonical activity. The reaction product is probably a thiol-containing compound: ultimately implicating novel thiol biology in plant-nematode parasitism.

### The detection of novel small thiols in syncytial feeding sites induced by nematodes

**Fig 6:**
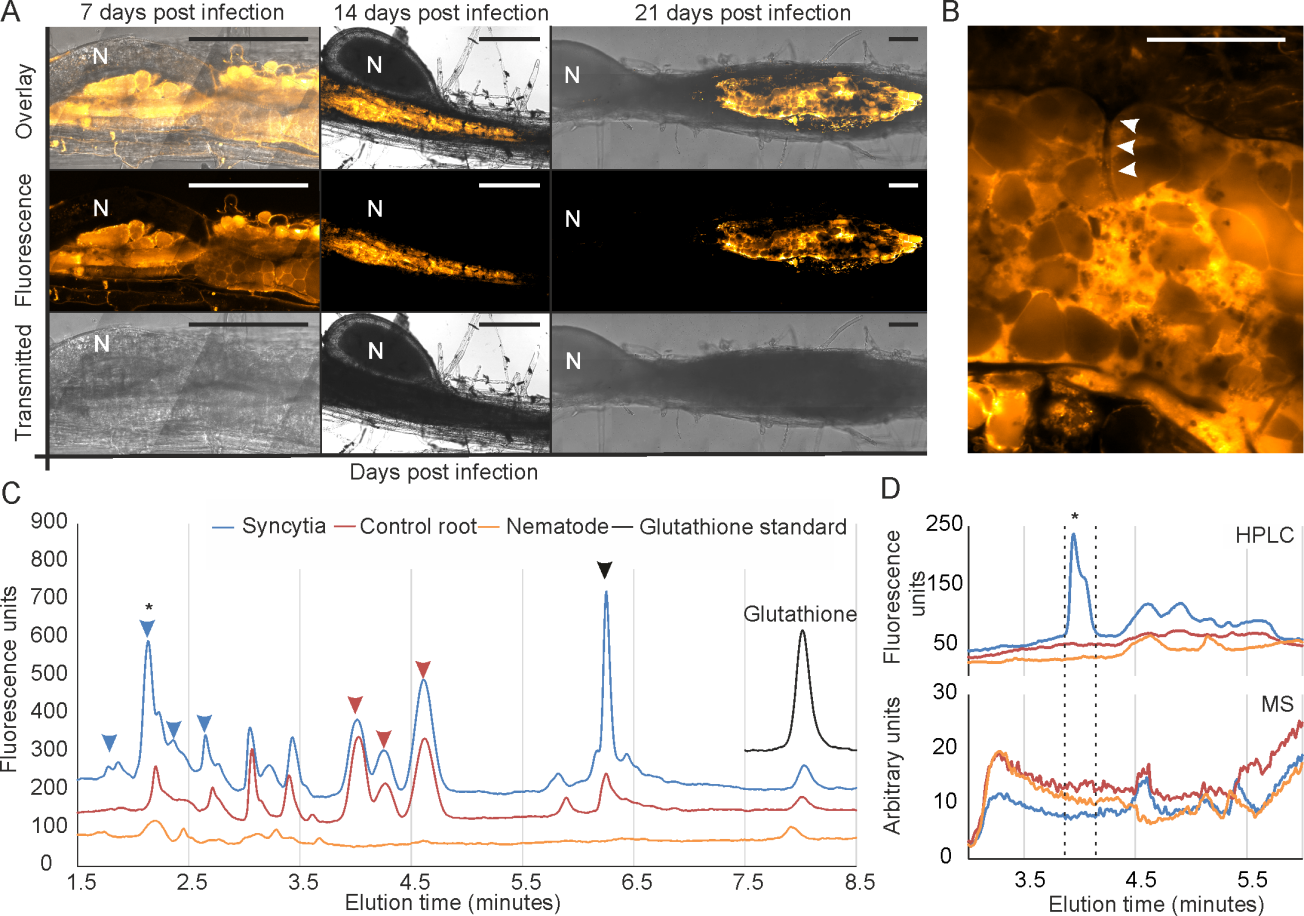
The detection of novel thiols in syncytial feeding sites induced by cyst nematodes. **A**) Free thiols, stained with ThiolTracker Violet, accumulate in the cytoplasm of syncytia induced by the cyst nematode *Heterodera schachtii* in *Arabidopsis* roots throughout the infection process (7, 14 and 21 days post infection). N indicates nematode. Scale bars = 100 μm. **B**) Optical cross section through the feeding site using the same stain. Arrows indicate partially dissolved cell wall. **C**) Analysis of low molecular weight thiols extracted from syncytia formed in potato by *Globodera pallida* (blue), control uninfected potato root (red), female nematodes (orange), and glutathione standard (black), by hydrophilic interaction liquid chromatography. Example plant-specific peaks are indicated with red arrows, syncytia-specific peaks with blue arrows, and a plant-specific peak increased in abundance in syncytia with a black arrow. **D**) The same sample used in panel C was separated at higher resolution using a shallower elution gradient. The peak at approximately 1.8 minutes in panel C (asterisk) corresponds to approximately 4 minutes in panel D (asterisk). This peak is absent in control roots, absent in nematode tissue, and highly abundant in syncytial feeding site material. No corresponding peak in the mass spectrum trace was identified (bottom).

To determine the extent of thiol involvement in syncytia induced by cyst nematodes, we initially employed a qualitative analysis to specifically stain and visualize free thiols in plant tissue during infection. We used the *Arabidopsis*-*Heterodera schachtii* pathosystem because the thin and transparent host roots are amenable to such studies, whereas those of other syncytial-forming nematodes (e.g. potato-*G*. *pallida)* are not. The *H*. *schachtii* transcriptome encodes a number of putatively secreted Clade 3 GS-like effectors (Fig 1). Using ThiolTracker Violet we discovered that thiols are abundant in, and largely localized to, the cytoplasm of syncytia induced by *H*. *schachtii*, throughout infection (Fig 6A). Following this support, short sections of infected potato root harbouring syncytia of *G*. *pallida* at 21 days post infection were collected, separated from their corresponding nematode, and both samples retained for analysis. Several hundred infection sites and nematodes were collected in this manner and pooled. Control uninfected adjacent root tissue was collected from the same plants. Low molecular weight (LMW) thiols present in the three samples were extracted, derivatized with mono-bromobimane and analysed by Hydrophilic Interaction Liquid Chromatography (HILIC, Fig 6C). We note that any increase in syncytial thiol abundance is not explained by an increase in glutathione, but a series of other LMW thiols (Fig 6C). Surprisingly, analysis of the area under each curve allowed us to roughly estimate that glutathione accounts for only approximately 2.5% of LMW thiols detected using this HPLC protocol in potato control roots, and 2.7% in syncytial segments. Furthermore, some of the novel LMW thiols are not present in uninfected potato root tissue, not present in nematodes, but only present in syncytia (Fig 6D). Although the detected novel thiols were recalcitrant to analysis by mass spectrometry (e.g. Fig 6D), their lower retention by HILIC allows us to infer they are more hydrophobic than glutathione.

### Disrupting redox regulation in syncytia is detrimental to nematode infection

Our demonstration of GS-like effectors and abundant thiols in syncytia, coupled with the importance of rbohD-dependent ROS production in syncytial development [37], point towards fine-tuned redox homeostasis during parasitism. We cannot target GS-like effectors in the nematode to disrupt redox homeostasis *in planta*. The lack of a transformation system precludes the generation of gene knockouts whilst the likely functional redundancy of GS-like effectors and their low sequence similarity would require the combined use of one RNAi construct per effector (n≈20) in order to achieve host-induced gene silencing. We therefore exploited the availability of *Arabidopsis* plants mutant in the first step of glutathione synthesis (*GSH-1* (*pad2-1))* and thus compromised in the endogenous glutathione portion of the syncytial thiol pool by approximately 50–80% [38].

**Fig 7:**
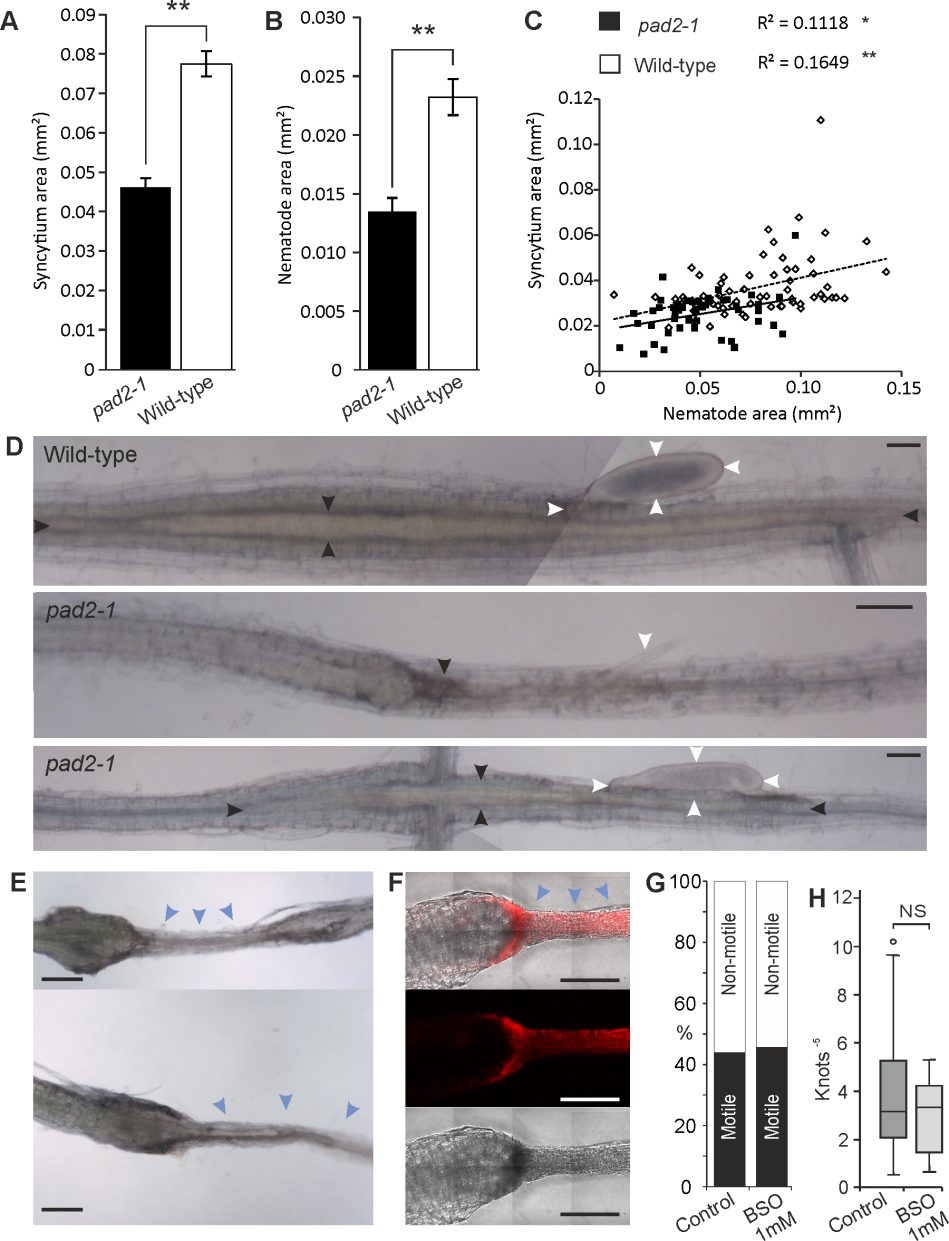
Impairing plant glutathione synthetic capacity is independently detrimental to syncytium and cyst nematode development. Both syncytia **(A)** and nematodes (**B)** are significantly smaller on *pad2-1* mutant *Arabidopsis* roots than on wild-type (Student’s T-test p ≤ 0.001, n = 82 and 147 for syncytia and nematodes respectively). Error bars indicate standard error of the mean. **(C)** In both *pad2-1* and wild-type roots, syncytium and nematode size significantly co-vary (Pearson’s correlation, p ≤ 0.05 and 0.001, n = 49 and 66 respectively), however the correlation is weak and most of the variation in nematode size (83–89%) is not explained by syncytium size. **(D)** Example images of unsuccessful nematode infection on *pad2-1* roots (middle) and successful infection on both *pad2-1* (bottom) and wild-type roots (top). Black and white arrows indicate syncytial and nematode boundaries respectively, blue arrows indicate an aborted syncytium and surrounding areas, scale bars indicate 100 μm. **E)** and **F)** In the presence of 1 mM BSO, syncytia are initiated but not properly maintained. Apoptosis, indicated by blue arrows and evidenced by propidium iodide staining (red), occurs in the local area of the syncytium (blue arrows) and spreads often non-distal to the site of infection; a similar, but more severe and frequent phenotype than observed in *pad2-1* roots. Scale bars indicate 100 μm. **(G)** and **(H)** J2 stage nematodes incubated for the normal root invasion period (48 hours) on water agar plates containing 1 mM BSO are unaffected in **(G)** mortality (n = 68) or **(H)** motility (Mann-Whitney U Test, p = 0.408 n = 20).

Both cyst nematode and syncytial development is significantly retarded at 10–12 days post infection in *pad2-1* compared to wild type (WT) plants (Student’s T-test p ≤ 0.001, n = 147 and 82 respectively, Fig 7A, 7B and 7D). Specifically, syncytia and nematodes supported by *pad2-1* plants are both on average ~50% the size of WT. However, despite that feeding site size and nematode size significantly co-vary in *pad2-1* and WT (Pearson’s correlation, p ≤ 0.05 and 0.001, n = 49 and 66 respectively), the correlation is weak: most of the variation in nematode size (83–89%) is not explained by syncytium size (Fig 7C). This suggests that the lack of plant glutathione synthesis is associated with at least two linked but largely unrelated processes during infection which individually contribute to nematode size, and feeding site size. Furthermore, in *pad2-1 Arabidopsis*, localised necrosis could often be seen surrounding syncytia, and aborted syncytia were common (Fig 7D and S6 Fig). Given that it is technically intractable to measure aborted syncytia and those obscured by localised necrosis, the effect may be even greater than we report.

In a parallel approach, we reduced glutathione synthetic capacity to approximately 16% of wild-type levels by the exogenous application of 1 mM L-buthionine-sulfoximine (BSO). BSO is an irreversible chemical inhibitor of the first step in the glutathione synthesis pathway [38]. At this dramatically reduced level, infective nematodes fail to maintain a compatible interaction. Under these conditions nematodes are able to penetrate root tissue, migrate to the vascular cylinder and initiate the formation of a syncytium. However, unlike the interaction with *pad2-1*, syncytia induced in plants treated with BSO are always aborted (Fig 7E). Necrosis occurs in the area of the developing syncytium, as evidenced by propidium iodide staining, and often spreads non-distal to the site of infection (Fig 7F). This phenotype is similar to, but more frequent and severe than, the necrosis seen in *pad2-1* plants. Necrotic patches on both *pad2-1* plants and BSO treated wild-type plants were associated only with nematode infection sites, while uninfected root was comparable to that of untreated wild-type plants, albeit with reduced proliferation (S7 Fig). We cannot rule out a direct effect of BSO on the nematodes during infection, however, infective stage nematodes incubated for 48 hours on water agar plates containing 1 mM BSO were largely unaffected in mortality or motility (Mann-Whitney U Test, p = 0.408; n = 20. Fig 7G and 7H).

## Discussion

Here we report a paradigm of effector gene birth for a plant pathogen of global economic importance. Cyst nematodes have exploited a series of gene gain events to redeploy glutathione synthetase-like enzymes during parasitism, within the syncytial feeding cell formed in the host root. We predict that the attributes acquired by GS-like paralogs that allow them to be deployed as effectors (e.g. DOG box promoter motif, change in spatial expression, change in temporal expression, gain of signal peptide) constitute a programme of effector evolution common to the genesis of other plant-parasitic nematode effectors from endogenous loci (e.g. SPRY-SECs [39]). The programme likely also applies to well-documented gene gain events in plant-parasitic nematodes (e.g. effectors derived from horizontal gene transfer events [33]), and perhaps even other pathosystems (e.g. aphids [40]). Studying effector gene birth may therefore contribute towards addressing a priority in the field: characterising effector repertoires of diverse plant-pathogens [41].

The structures of the GS-like effectors led us to employ non-biased approaches to measure and analyse thiol biology during parasitism. Ultimately this resulted in the unexpected discovery of a range of novel thiols associated with the nematode feeding sites in host roots. Whilst cysteine, γ-EC and glutathione are major LMW thiols common to both plants and animals, analysis here of potato, and previously of other species [42], reveals a diverse array of unidentified LMW thiols in plants. Many of the very large number of undescribed compounds (~200) discovered in the Arabidopsis sulfur metabolome [43] could be LMW thiols, representing a pool of potential novel cysteine-containing substrates for the nematode GS-like effectors.

The thiol moiety, a nucleophile occurring predominantly in cysteine residues, is one of the most chemically reactive groups in biological systems and plays a major role in maintenance of cellular redox homeostasis. In most other plant-pathogen interactions described to date, the strong nucleophile glutathione is a negative regulator of pathogenicity [18–22, 24, 44, 45]. For example, the Arabidopsis *pad2-1* mutant that has reduced glutathione content is more susceptible to a range of pathogens including *Pseudomonas syringae* and *Phytophthora brassicae* [24], whilst increased glutathione enhances plant defence responses [21]. The RipAY effector from the bacterial pathogen *Ralstonia solanacearum* has recently been shown to specifically target host glutathione in order to promote pathogenicity. It acts as a γ–glutamyl cyclotransferase to deplete intracellular glutathione, further emphasising the important role of this thiol in plant immunity [46–48]. A notable exception is the discovery that homoglutathione deficiency impairs root-knot nematode development in *Medicago truncatula* [49]. Here we show that Arabidopsis plants deficient in endogenous glutathione synthesis are less susceptible to cyst nematodes. This initially would thus seem unsurprising, however the necrosis and aborted feeding sites that result from depletion of glutathione during cyst nematode parasitism are not apparent for root-knot nematodes [49], suggesting different roles for glutathione in the two interactions. It is also important to note that cyst and root-knot nematodes have independent evolutionary origins of sedentary endo-parasitism, have almost no overlap in effector complement [39], and produce feeding sites that are different in structure and ontogeny [50]. Taken together, we cannot draw clear parallels between these superficially similar discoveries in two dissimilar pathosystems. Nevertheless, we show that plant-derived glutathione is a positive regulator of cyst nematode parasitism.

Interestingly, the expansion of Clade 2 GS enzymes, which preceded that of the GS-like effectors, is common to cyst nematodes, root-knot nematodes, and indeed all endoparasitic nematode species within the Tylenchida (including those that do not establish a feeding site within the host). Clade 2 GS enzymes do not encode a secretion signal and are expressed in the intestine. While the Clade 2 GS enzyme tested clearly has a slower rate of canonical enzyme activity than the Clade 1 GS, it can nevertheless synthesise glutathione: It has a high affinity for the γ-EC dipeptide substrate, and a preference for glycine as the terminal amino acid despite a lack of conservation in the two residues that apparently contribute to this specificity. While we cannot rule out the existence of other substrates, Clade 2 GS-like enzymes have retained canonical activity. Many Clade 2 GS share a short and somewhat variable C-terminal extension that is absent from all other clades and is not recognised as a canonical signal by TargetP. We can assume that the conservation of this C-terminal extension implies the existence of some functional constraints that remain to be elucidated.

In conclusion, we implicate a positive role for novel nucleophiles in parasitism of cyst nematodes. We show three discoveries that are functionally independent but grouped under the banner of redox homeostasis in the plant cell, 1) Nematode-derived GS-like effectors likely accept a thiol substrate, but do not produce glutathione (Figs 3,4 and 5); 2) Syncytia are abundant in novel thiols of unknown origin (Fig 6); and 3) Plant-derived glutathione is a positive regulator of cyst nematode parasitism (Fig 7). In contrast to this, it was shown that rbohD-dependent ROS production is also integral to feeding site development, and is necessary to limit cell death and promote cyst nematode parasitism [37]. Taken together, these data collectively support the hypothesis that nematode-induced syncytia operate within a narrow redox “Goldilocks zone”. The focus of future research will be to determine if any of these discoveries are dependent on one another biochemically, what is the cross talk between the various aspects of redox regulation, and how, together, they contribute to parasitism.

## Materials and methods

### Molecular phylogenetic analyses

RNAseq reads for *Heterodera avenae* [51], *H*. *schachtii* (doi:10.5061/dryad.7vd0160.) and *Rotylenchulus reniformis* [52] were trimmed according to previously described methods except that HEADCROP was set to 11, 10 and 12 respectively [26]. Trimmed reads were assembled into *de novo* transcriptomes using the Trinity pipeline [53] with a minimum Kmer coverage of 2. Proteins were predicted using the Trinity wrapper scripts for transdecoder using the Pfam A and B library. GS genes were predicted from the assemblies generated above, existing transcriptome assemblies for *Nacobbus aberrans* [26], *Longidorus elongatus* [54], and *Pratylenchus penetrans* [55] and existing genome assemblies for *Globodera rostochiensis* [28], *Globodera pallida* [12], *R*. *reniformis* (doi:10.5061/dryad.7vd0160.), *Meloidogyne incognita* [25, 56], *Bursaphelenchus xylophilus* [57], and *Caenorhabditis elegans* [58] using Pfam (PF03917/PF03199). For *R*. *reniformis*, additional GS-like sequences were identified in the genome and transcriptome by sequence similarity searches (BLAST v 2.4.0; [59]) using all *G*. *pallida* GS-like proteins as queries. The results of these two identification pipelines were merged and a list of unique *R*. *reniformis* GS-like sequences was compiled from both the genome and transcriptome.

Drastically truncated sequences identified following alignment of all encoded proteins (MUSCLE v3.8.3.1; [60]), were removed or, for genomic predictions, manually curated where possible based on transcript coverage and/or homology. Any incomplete genomic predictions that could not be corrected due to missing sequence were also removed from further analysis. For *G*. *pallida*, upstream regions (2 kb) of all genomic predictions were analysed for the presence of additional exons that could encode signal peptides. The majority of *G*. *pallida* full-length coding regions, including all those where manual curation conflicted with the original gene prediction, were amplified using Phusion polymerase from cDNA prepared from early parasitic stage nematodes. Primers used are detailed in S5 Table. The number of predicted *G*. *pallida* GS genes that encoded a signal peptide increased following the manual curation and subsequent cloning. The amino acid sequences of those genes from all species remaining after curation were aligned and refined using MUSCLE v3.8.3.1 [60]. The alignment was trimmed using TrimAL (-gappyout) [61] and subject to model selection (WAG+GAMMA with invariable sites) and Bayesian phylogeny construction (Mr Bayes) with two million five hundred thousand generations, a sample frequency of 10%, and a burn in rate of 30% carried out in TOPALi v2.5 [62]. The phylogeny was out-group routed by the Clade containing the *C*. *elegans* and *L*. *elongatus* sequences [63] using FigTree v1.4 (http://tree.bio.ed.ac.uk/software/figtree/). Where available (*G*. *rostochiensis*, *G*. *pallida*, *H*. *avenae*, *R*. *reniformis*, *H*. *schachtii*, and *N*. *aberrans*), RNAseq reads were mapped back to either the relevant assembly, or in the case of *G*. *rostochiensis* and *R*. *reniformis* the manually curated GS-like transcripts, and normalized expression values (TMM) were calculated using the Trinity wrapper scripts for RSEM and EdgeR using default parameters. Expression fold change was calculated by dividing average normalized expression at parasitic life stages (any stages recovered from roots) by that of non-parasitic (eggs, second stage juveniles and, for *G*. *pallida*, adult males). Multiple biological replicates were available for *G*. *pallida* (two), *G*. *rostochiensis* (two), *R*. *reniformis* (three) and *N*. *aberrans* (three). In depth transcriptional analyses across the life cycle of *G*. *pallida* were performed using normalized expression values available [12]. Signal peptides were predicted using SignalP v4.0 [64].

### *In situ* hybridisation

For the cyst nematodes *G*. *pallida* and *G*. *rostochiensis*, *in situ* hybridisation was carried out on 3^rd^ (J3) and 4^th^ (J4) stage juveniles and young adult females extracted from roots of potato (*Solanum tuberosum*) according to previously described methods [28]. For sedentary stage female *R*. *reniformis* nematodes extracted from roots of cotton the same methods were followed except that the proteinase K treatment was reduced to 1 hour at room temperature. Single-stranded 100–200 base pair DNA probes corresponding to sequences of interest were prepared as described [24] using the oligonucleotide primers detailed in S5 Table. For each gene of interest, an equivalent sense-strand probe acted as a negative control. More than 100 individual nematodes were examined for each probe and the results presented are representative of the staining patterns observed.

### Immunolocalisation of GS-like effector protein

Purified, recombinant Gpa-GSS17 protein was used to raise a polyclonal antibody in rabbit that was affinity-purified against the original antigen by Cambridge Research Biochemicals (Billingham, UK). For detection of the protein in nematodes, mixed parasitic stages of *G*. *pallida* were recovered from potato roots, fixed, cut, permeabilized and dehydrated as for *in situ* hybridisation. Rehydrated nematodes were washed with maleic acid buffer then incubated in the same buffer containing 1% blocking reagent (Roche) for 30 mins at room temperature. Following an overnight incubation at 4°C in blocking buffer containing Gpa-GSS17 antibody at a dilution of 1 in 200, nematodes were washed, reblocked for 30 mins and incubated with FITC-conjugated goat anti-rabbit 2^o^ antibody (Sigma) at a dilution of 1 in 200 for 2h at room temperature. After three washes in maleic acid buffer containing 0.01% Tween-20, nematodes were resuspended in anti-fadent (PBS/glycerol; Citifluor) and visualized using a Leica DMRB microscope with GFP filter set. The experiment was carried out on separate occasions with two batches of fixed nematodes and >100 individual nematodes were observed on each occasion. Images were captured with a QIcam camera (QImaging) and Q-Capture software. The images presented are representative of all those that displayed hybridisation of the antibody. Control nematodes were processed in the same manner with the omission of primary antibody.

For detection of Gpa-GSS17 in syncytia, lengths of potato root 14 days post infection with J2 of *G*. *pallida* were fixed in 4% paraformaldehyde in PEM buffer (50 mM PIPES, 10 mM EGTA, 10 mM MgSO_4_ pH 6.9) for 3 days at 4°C. Samples were dehydrated, resin embedded, sectioned and applied to microscope slides according to Davies *et al*. [65] Transverse sections through the nematode feeding site were blocked with 5% milk powder in PBS for 3 h, then incubated in primary Gpa-GSS17 antibody at a dilution of 1 in 50 in 0.5% milk powder/PBS overnight at 4°C. After washing in PBS, primary antibody was detected with a FITC-conjugated anti-rabbit secondary antibody at a dilution of 1 in 100. At least 20 sections through each of three separate syncytia were analysed. Control sections were treated identically except for the omission of primary antibody. Plant cell walls were stained by incubation in Calcofluor-White (1 mg/ml) for 5 mins, followed by copious washes with PBS. Antibody localisation was visualized and recorded as described above for nematodes.

### Cloning, heterologous expression and protein purification

All GS-like genes analysed were cloned (without their predicted signal peptide if appropriate (SignalP v4.0)) into the pOPINS3C vector [66] in frame with an N-terminal poly-Histidine tag, a SUMO chaperone to promote protein solubility, and a 3C protease cleavage site (S5 Table). GS-like genes were expressed in, and their encoded proteins purified from, *E*. *coli* strain Shuffle to allow disulphide bond formation [67]. Cell cultures were grown in Luria Bertani media at 30°C until an optical density of 0.6–0.8 at A_600_ was reached. Cell cultures were cooled to 18°C and expression of the GS-like proteins of interest induced with addition of IPTG to a final concentration of 1 mM. Proteins were allowed to express for 14 hours at 18°C and the cells were collected by centrifugation and lysed immediately. Cell pellets were re-suspended in 50 mM Tris-HCl, 500 mM NaCl, 50 mM glycine, 5% (v/v) glycerol and 20 mM imidazole, pH 8.0 with the addition of one EDTA-free protease inhibitor tablet per 50 ml, and lysed by sonication. Cell lysate was clarified by centrifugation and applied to a 5 ml Ni^2+^-NTA column on an AKTA Xpress. His-tagged proteins were step eluted in resuspension buffer + 500 mM imidazole and injected onto a Superdex 200 26/60 gel filtration column equilibrated to 20 mM HEPES and 0.15 M NaCl, pH 7.5. Fractions containing the protein of interest were pooled, concentrated to ~5 ml, and the His+SUMO tag cleaved by overnight digestion with 3C protease at 4°C at a ratio of 100:1 (protein:protease). Mature GS-like proteins were separated from the His+SUMO tag by passing the solution over a 5 ml Ni^2+^-NTA column and injecting the flow-through onto a Superdex 200 26/60 gel filtration column. The concentration of each protein was measured by direct detection of the peptide bond (Direct detect), and protein aliquots were stored at -80°C until needed.

### Enzymology

All enzyme assays were carried out at 30°C in a typical reaction buffer of 100 mM HEPES (pH 7.5), 20 mM MgCl_2_, and 5 mM dithiothreitol, with the addition of ATP, γ-EC, and glycine at varying concentrations. The hydrolysis of ATP in the presence of each protein, relative to the control, was used as a molar equivalent proxy for the production of glutathione, and measured by detection of free phosphate using malachite green absorbance at 630 nm. After determining the linear range of the reaction over time, pkat values of each protein in the presence of 1 mM γ-EC, 2.5 mM ATP and 100 mM glycine were measured in triplicate, and compared to a standard curve of free phosphate. To estimate the Michaelis-Menten kinetics of those enzymes with a rate appreciably above their negative control, γ-EC was varied in serial dilution and data analysis carried out in Sigmaplot. Experiments to explore the substrate specificity at the terminal amino acid of all GS-like enzymes were carried out using 2.5 mM ATP and 1 mM γ-EC with the following amino acids at 100 mM: glycine, β-alanine, D-alanine, GABA, AABA, diaminopropionic acid, D-serine).

### Crystallography

Purified GS-like proteins were concentrated to between 5 and 10 mg/ml in 20 mM Tris (pH 7,5) and 200 mM NaCl. Sitting drop vapour diffusion crystallization experiments at 20°C were carried out in 96 well format using an OryxNano robot. Gpa-GS22 crystallized readily in a number of conditions in several screens at 5 mg/ml final concentration. Screen JCSG condition D10, was optimized to produce crystals in 0.2 M tri-methylamine N-oxide, 0.1 M Tris pH 9 and 20% w/v PEG 2000 MME. Several crystals were transferred to cryoprotectant (mother liquor with the addition 20% ethylene glycol final concentration) and frozen in a loop in liquid nitrogen. X-ray diffraction data were collected at the Diamond light source beamlines i04-1, processed using the xia2 pipeline [68], and the structure was solved by molecular replacement with 3KAL and named Gpa-GSS22-apo. The submitted structure was obtained through an iterative process of manual building and refinement using COOT [69] and REFMAC5 respectively. Tools of COOT and MOLPROBITY [70] were used for structure validation. Further, Gpa-GSS22, at a final concentration of 2.5 mg/ml, crystallized in the same condition with the addition of ADP (2.5 mM), MgCl_2_ (5 mM) and glutathione (2.5 mM). Diffraction data (beamline i02), structure solution, refinement, and validation were carried out as for Gpa-GSS22-apo, using the solved structure of Gpa-GSS22-apo in molecular replacement. Protein crystals for Gpa-GSS30 were obtained in JCSG E1 (0.2 M magnesium formate di-hydrate and 20% PEG 3350) and the structure solved/refined/validated as described above, using Gpa-GS22-apo for molecular replacement. Protein crystals for St-GSS1 were obtained by optimisation of JCSG D9 (1.4 M D-malic acid) with the addition of 2.5 mM γ-EC, 2.5 mM ADP, 5 mM MgCl_2_ and St-GSS1 at 3.75 mg/ml final concentration. The structure was solved/refined/validated as described above, using molecular replacement with the structure of hGS of *Glycine max* (3KAL).

### Analysis of thiols in nematode-induced feeding sites

Intra-cellular free thiols were visualized in syncytia produced by *H*. *schachtii* 7, 14 and 21 days post infection by incubating lengths of *Arabidopsis* root in 5 mM ThiolTracker Violet (Life Technologies) for 2 hours at room temperature. This fluorescent dye reacts with any reduced thiols, including glutathione, in live cells. Samples were rinsed twice in thiol-free PBS (Life Technologies) prior to imaging with a Zeiss LSM700 confocal microscope (excitation at 405 nm and collection from 410–500 nm).

For thiol analysis, roots of potato plants cv Desiree were harvested 28 days after planting tubers into sandy loam soil infested with cysts of *G*. *pallida*. Plants were grown at 20°C in a glasshouse with a 16h:8h light:dark cycle. Roots were washed to remove adhering soil particles and maintained in water while infection sites were identified using a stereobinocular microscope. Young adult female nematodes at approximately 21 days post infection were carefully removed intact from the root and collected. The length of root harbouring the syncytium (3–4 mm) was excised and collected separately. Equivalent, uninfected root lengths were harvested from the same root system. A total of ~200 feeding sites/nematodes/control root sections were amassed on each experimental occasion. Tissue in 1.5 ml tubes was flash frozen in liquid N_2_ and stored at -70°C until use. Samples were thawed in 130 ul of 0.1 M HCL and homogenized with a micro pestle. The cell lysate was clarified by centrifugation twice (10 minutes, 4°C, 20,000 RPM) and 50 μl was removed for derivatisation of thiols by addition of 1.5 μl 1 M DTT, 1.5 μl Mono-bromobimane and 45 μl 1 M CHES pH 9. The reaction was incubated at room temperature for 20 minutes, and stopped by the addition of 50 μl of 50% acetic acid. Samples were analysed using a Shimadzu LC/MS system comprising Nexera UHPLC binary pumps and autosampler, Prominence fluorescence and UV diode array detectors and LCMS-2020 single quadrupole mass spectrometer with ESI/APCI dual ion source. Two microliters of each sample was injected onto a Accucore 150-Amide–HILIC 100mm x 2.1mm column held at 25°C, and low molecular weight thiols were separated by a gradient of 0.1% v/v formic acid (Buffer A) and acetonitrile (Buffer B): a linear gradient from 90–85% B over 6 minutes followed by 85% B for 2 minutes, 60% B for 1.5 minutes, and 90% B for 2.5 minutes. Samples were eluted at 0.4 ml/minute and mono-bromobimane derivatives were detected by fluorescence at excitation/emission 397/480 nm. Mass spectra were collected continuously using a DL temperature of 250°C, a nebulizing gas flow rate of 1.5 L/min, a heat block temperature of 400°C, a drying gas flow rate of 15 L/min and in both ESI and APCI ionisation mode. Mass spectra were collected in scan mode with both +ve (4 kV) and–ve (-3.5 kV) across a range of 100–1200 Da. To determine the elution time of glutathione under these conditions, samples were spiked with 1 μl of 10 mM glutathione standard (Sigma) after clarification of the HCL extract by centrifugation.

### Arabidopsis infection assays

Surface sterilised *Arabidopsis thaliana* (Col-0) or *pad2-1* seeds were grown for 15 days on 9 cm vertical plates containing ½ strength Murashige and Skoog medium supplemented with 10 g/l sucrose and 1% Phytagel (½ MS10). Hatched second stage juveniles (J2) of *H*. *schachtii* were sterilised [71] and resuspended at a concentration of approximately one nematode/μl. 20 μl of suspension was pipetted onto each of two root points per plant with two plants per plate. Infection points were covered with GF/A paper (Whatman) for two days to facilitate invasion. At 10–12 days post infection, nematode (excluding un-emerged adult males) and syncytium size were estimated from the projected cross-section as viewed under a microscope (Olympus BH2) and measured using Image-Pro Analyser v7 (MediaCybernetics). Lengths of root containing aborted syncytia were incubated in propidium iodide (10 μg/ml) for 5 minutes at room temperature, washed twice in PBS, and imaged with a Zeiss LSM700 (excitation at 488 nm and collection from 590–700 nm). For L-Buthionine-Sulfoximine (BSO) treatment, wild-type seedlings were transferred to ½ MS10 plates containing 1 mM BSO two days prior to infection. BSO toxicity to nematodes over the course of the invasion period was tested separately by incubating J2s on water agar plates with or without 1 mM BSO in the absence of any plants. Nematode mortality was scored by observing each nematode for 5–10 seconds to record movement. Nematode motility was assessed by measuring nematode speed over 2 minutes using the ImageJ (http://imagej.nih.gov/ij/) plugin wrMTrck (http://www.phage.dk/plugins/wrmtrck.html).

## Supporting information

S1 Fig C-terminal extension associated with Clade2 GS that is absent from all Clade 1 and Clade 3 GS. A) Alignment of a short C-terminal extension associated with Clade 2 GS coloured according to hydrophobicity. B) The consensus sequence of the Short C-terminal extension of unknown significance.
(TIF)

S2 Fig Detailed life stage-specific transcriptional profile of *Globodera pallida* GS-like genes from each of the three clades. Average normalised expression values for two biological replicates of RNAseq (12) are plotted for each life stage (egg, second-stage juvenile (J2) parasitic stages at 7, 14, 21, 28 and 35 days post infection (DPI) and non-parasitic adult males). GS genes are colour coded by Clade. (Clade 1 = Black, Clade 2 = Grey, Clade 3 = Red).
(TIF)

S3 Fig In situ hybridisation of *Globodera pallida* GS-like genes from each of the three clades. *In situ* hybridisation of digoxygenin-labelled DNA probes to GS transcripts in sedentary female or male stages of *G*. *pallida* cyst nematodes. For each row, left shows positive staining pattern with probe to target sense mRNA, right shows negative control staining pattern with probe to target anti-sense mRNA. Clade 1 and 2 GS-like genes are expressed (dark staining) throughout the punctate digestive system and expression is absent from the gland cells. Three putatively secreted GS genes from Clade 3 are expressed in the large dorsal pharyngeal gland cell posterior to the metacorpal bulb and anterior to the punctate digestive system.
(TIF)

S4 Fig Analytical gel filtration of selected purified GS-like proteins is consistent with obligate homo-dimer formation. Selected purified GS-like proteins migrate on an S200 analytical gel filtration column between the 150 kDa and 75 kDa protein standards, at an approximate molecular mass consistent with obligate homodimer formation.
(TIF)

S5 Fig Substrate specificity of selected purified GS-like enzymes. **A)** Alignment of substrate binding loop in GS enzymes from plant, animal and fungal kingdom compared to homoGlutathione synthetase of *Glycine max* (hGS). Two adjacent and highly conserved alanine residues are substituted in hGS to leucine and proline, and contribute to substrate specificity. Residues in these positions are different but invariable in Tylenchoidea Clade 1, variable in Clade 2, and highly variable in Clade 3. **B)** Analysing the terminal amino acid preference of purified GS-like proteins shows that St-GSS1, Gpa-GSS1 and Gpa-GSS5 have a preference for canonical substrates. None of the Clade 3 GS-like effectors show an appreciable rate of turnover with any of the terminal amino acids tested. **C)** The K_m_ for γ-EC for canonical GS enzymes. Despite a lower absolute rate, the Clade 2 Gpa-GSS5 has a higher affinity for γ-EC than Gpa-GSS1.
(TIF)

S6 Fig Localised necrosis surrounding syncytia formed in *pad2-1* Arabidopsis. Nematodes (white arrows) and syncytia (black arrows) can be seen 10–12 days post infection displaying varying degrees of necrosis surrounding the syncytia and poor nematode development. It is not possible to measure the size of syncytia largely obscured by necrosis.
(TIF)

S7 Fig Phenotypic comparison of wild type, *pad2-1* and BSO treated *Arabidopsis*. Root phenotype comparison between *Arabidopsis* wild-type, *pad2-1* mutant, and wild-type plants treated with 1 mM BSO.
(TIF)

S1 Table DOG box enrichment by clade. (XLSX)

S2 Table Glutathione synthetic activity assay. (XLSX)

S3 Table Structure statistics table. (XLSX)

S4 Table Structure-guided comparison of residues in ADP binding pocket and substrate binding pocket of GS, and GS-like effectors. (XLSX)

S5 Table Primers. (XLSX)

S6 Table Underlying numerical data for Fig 1 graph adjacent to phylogeny. (XLSX)

S7 Table Underlying numerical data for Fig 3 graph of enzyme rates with buffer controls. (XLSX)

S8 Table Underlying numerical data for Fig 7. (XLSX)
