# Supplementary figures and images for: Effector gene birth in plant parasitic nematodes: Neofunctionalization of a housekeeping glutathione synthetase gene
Source: PLoS Genet. 2018 Apr 11;14(4):e1007310. doi: 10.1371/journal.pgen.1007310 (PMC5919673; doi:10.1371/journal.pgen.1007310)

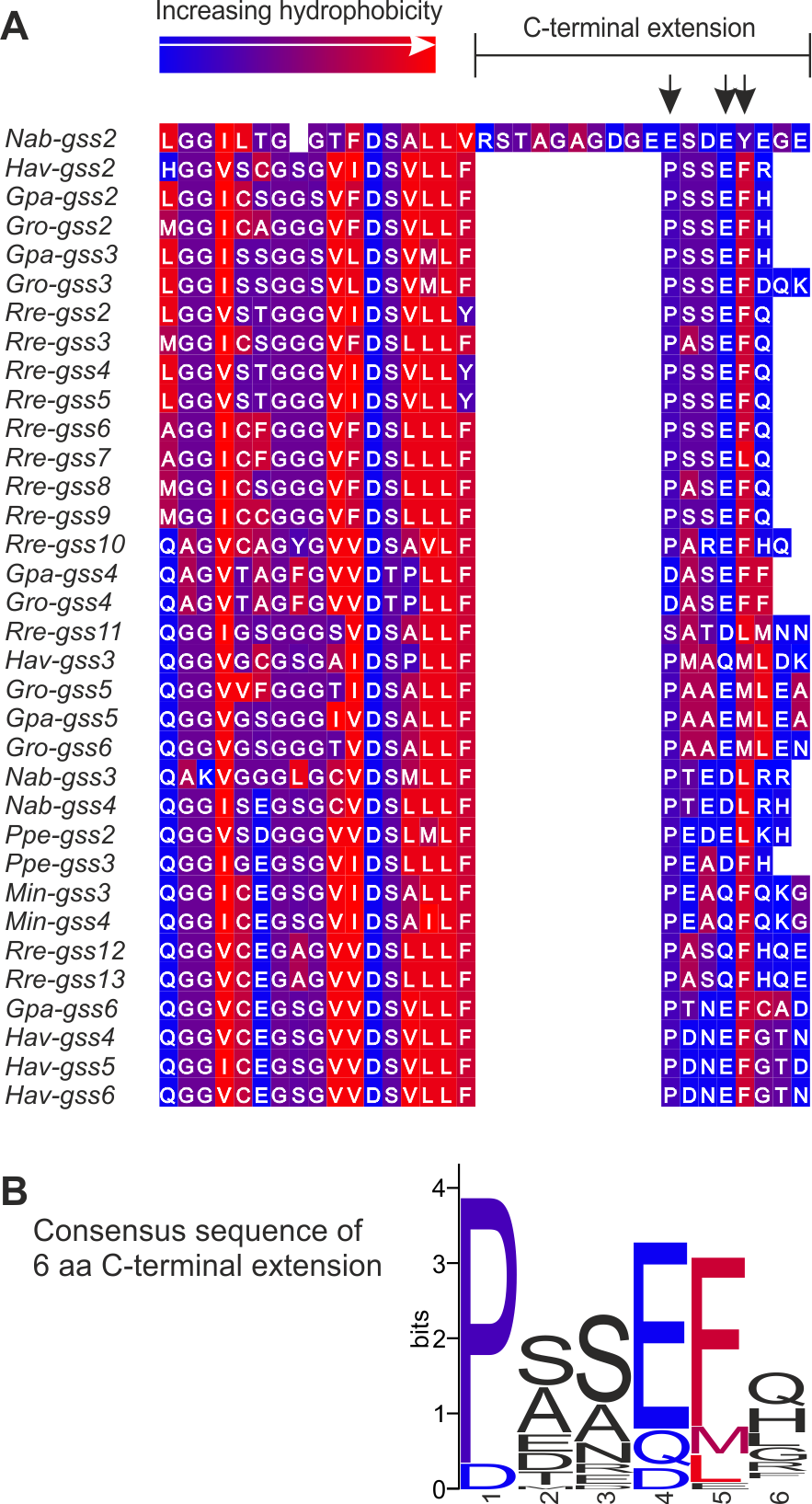

Supplement: S1 Fig — A) Alignment of a short C-terminal extension associated with Clade 2 GS coloured according to hydrophobicity. B) The consensus sequence of the Short C-terminal extension of unknown significance. (TIF) [file pgen.1007310.s001.tif]

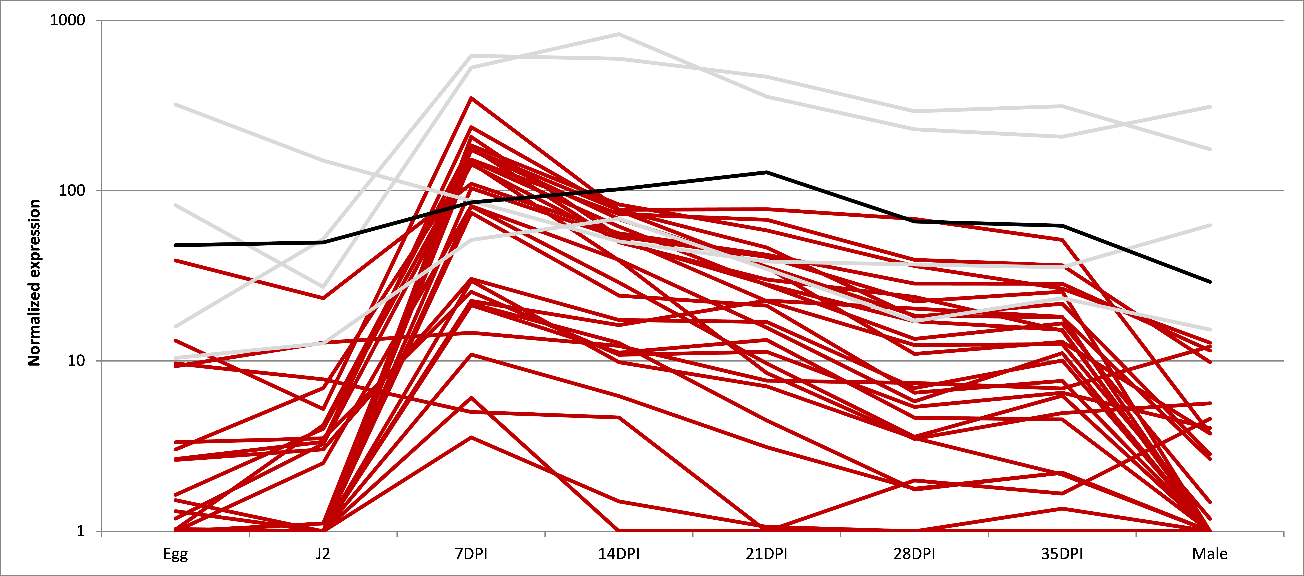

Supplement: S2 Fig — Average normalised expression values for two biological replicates of RNAseq (12) are plotted for each life stage (egg, second-stage juvenile (J2) parasitic stages at 7, 14, 21, 28 and 35 days post infection (DPI) and non-parasitic adult males). GS genes are colour coded by Clade. (Clade 1 = Black, Clade 2 = Grey, Clade 3 = Red). (TIF) [file pgen.1007310.s002.tif]

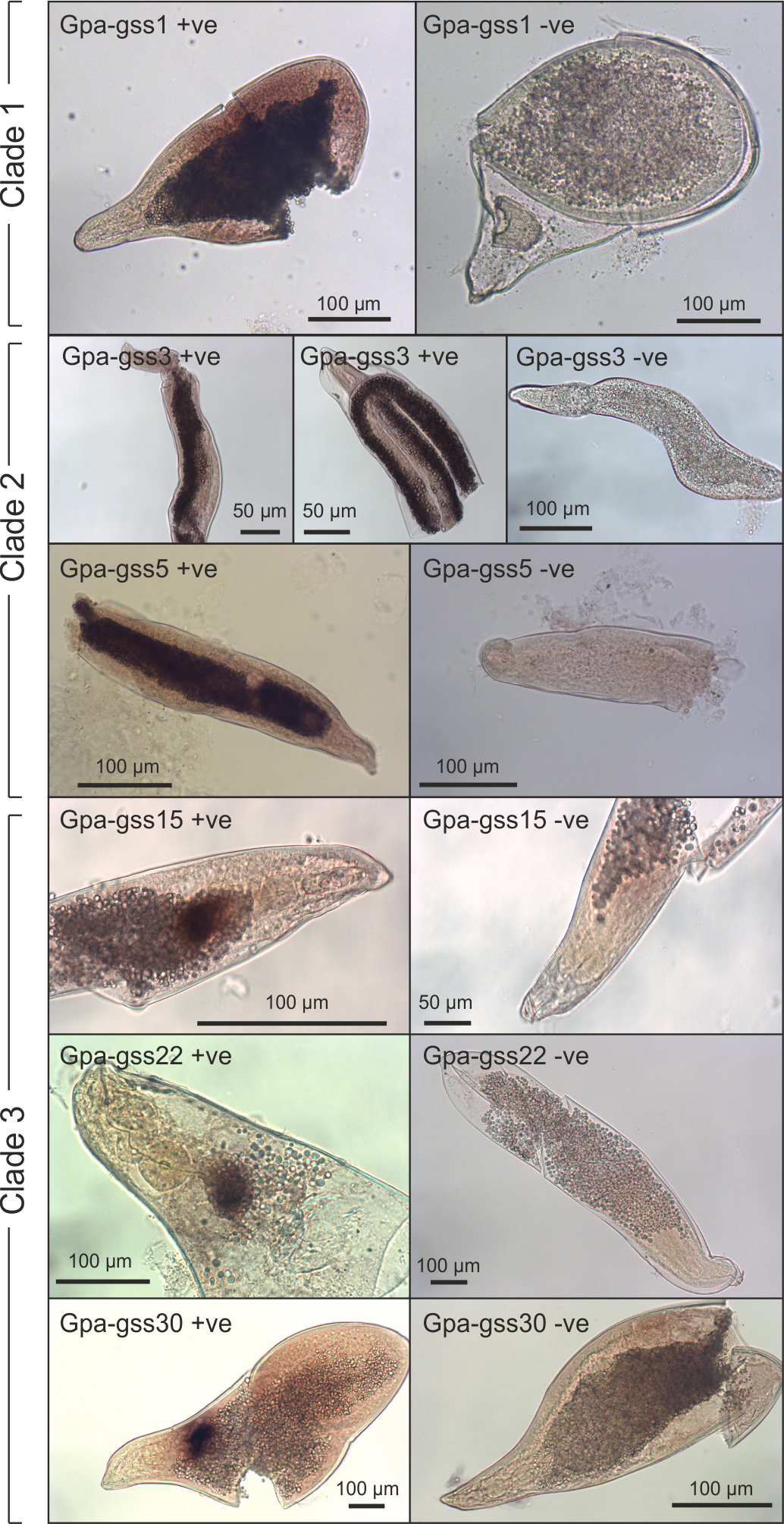

Supplement: S3 Fig — In situ hybridisation of digoxygenin-labelled DNA probes to GS transcripts in sedentary female or male stages of G. pallida cyst nematodes. For each row, left shows positive staining pattern with probe to target sense mRNA, right shows negative control staining pattern with probe to target anti-sense mRNA. Clade 1 and 2 GS-like genes are expressed (dark staining) throughout the punctate digestive system and expression is absent from the gland cells. Three putatively secreted GS genes from Clade 3 are expressed in the large dorsal pharyngeal gland cell posterior to the metacorpal bulb and anterior to the punctate digestive system. (TIF) [file pgen.1007310.s003.tif]

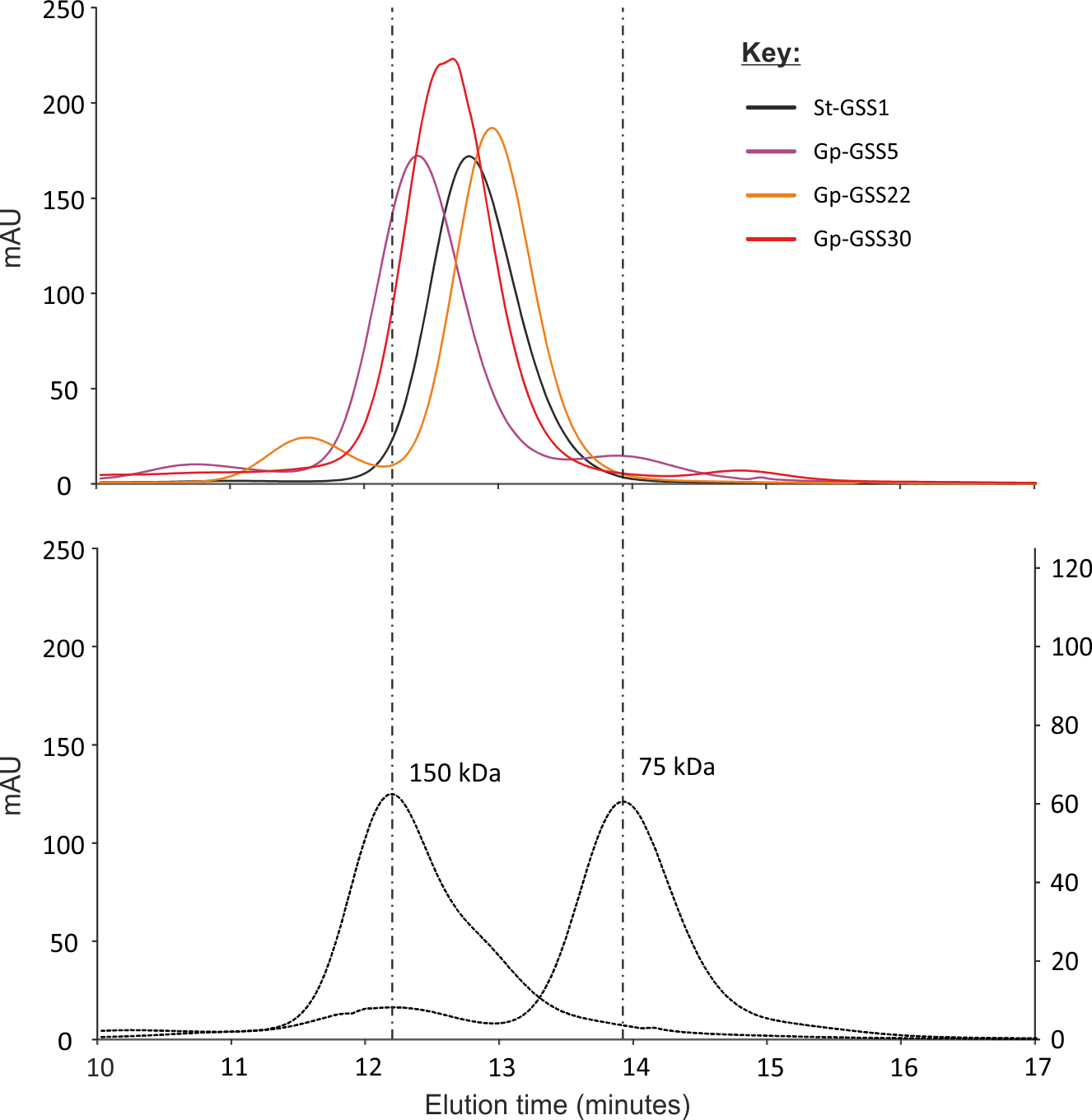

Supplement: S4 Fig — Selected purified GS-like proteins migrate on an S200 analytical gel filtration column between the 150 kDa and 75 kDa protein standards, at an approximate molecular mass consistent with obligate homodimer formation. (TIF) [file pgen.1007310.s004.tif]

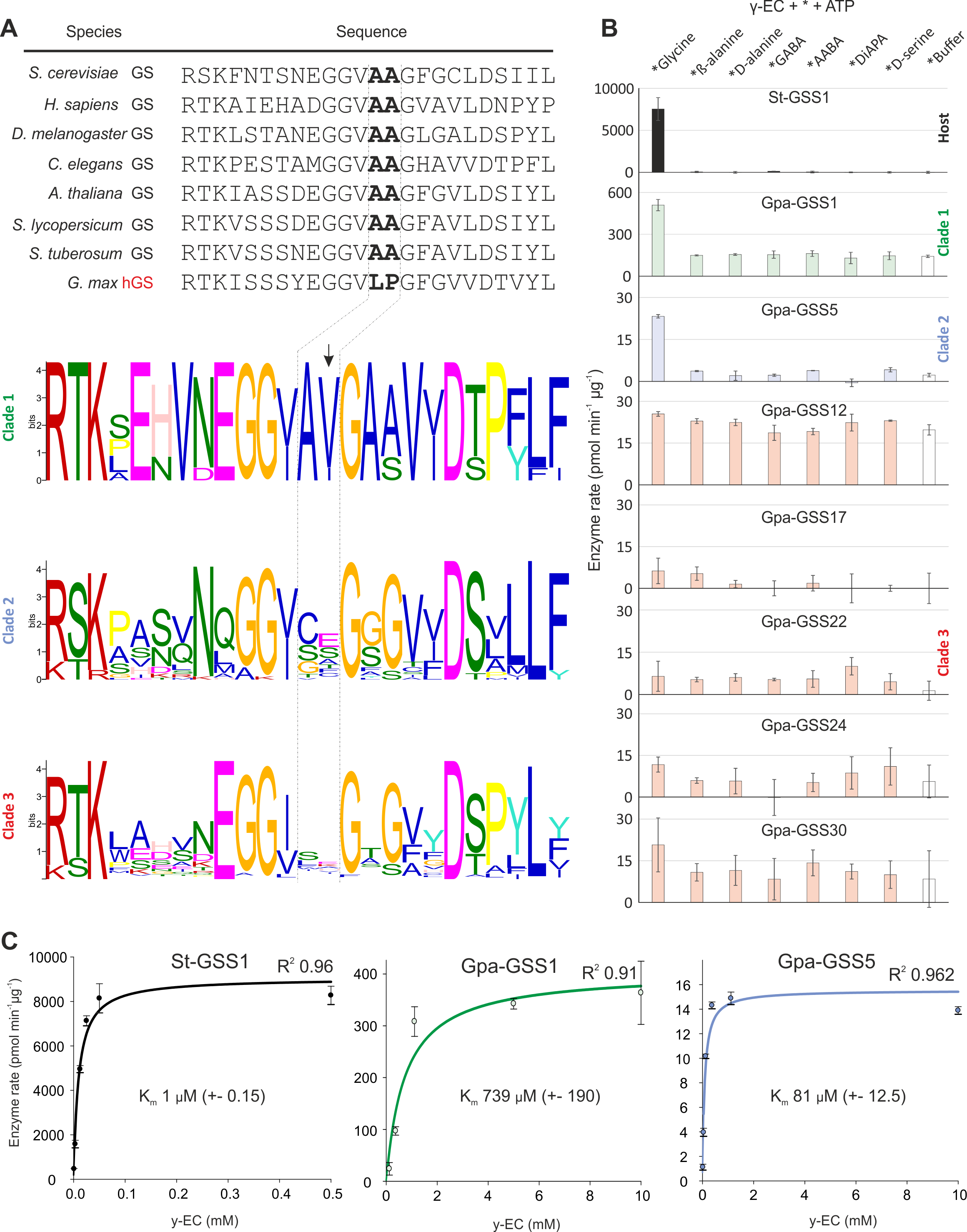

Supplement: S5 Fig — A) Alignment of substrate binding loop in GS enzymes from plant, animal and fungal kingdom compared to homoGlutathione synthetase of Glycine max (hGS). Two adjacent and highly conserved alanine residues are substituted in hGS to leucine and proline, and contribute to substrate specificity. Residues in these positions are different but invariable in Tylenchoidea Clade 1, variable in Clade 2, and highly variable in Clade 3. B) Analysing the terminal amino acid preference of purified GS-like proteins shows that St-GSS1, Gpa-GSS1 and Gpa-GSS5 have a preference for canonical substrates. None of the Clade 3 GS-like effectors show an appreciable rate of turnover with any of the terminal amino acids tested. C) The Km for γ-EC for canonical GS enzymes. Despite a lower absolute rate, the Clade 2 Gpa-GSS5 has a higher affinity for γ-EC than Gpa-GSS1. (TIF) [file pgen.1007310.s005.tif]

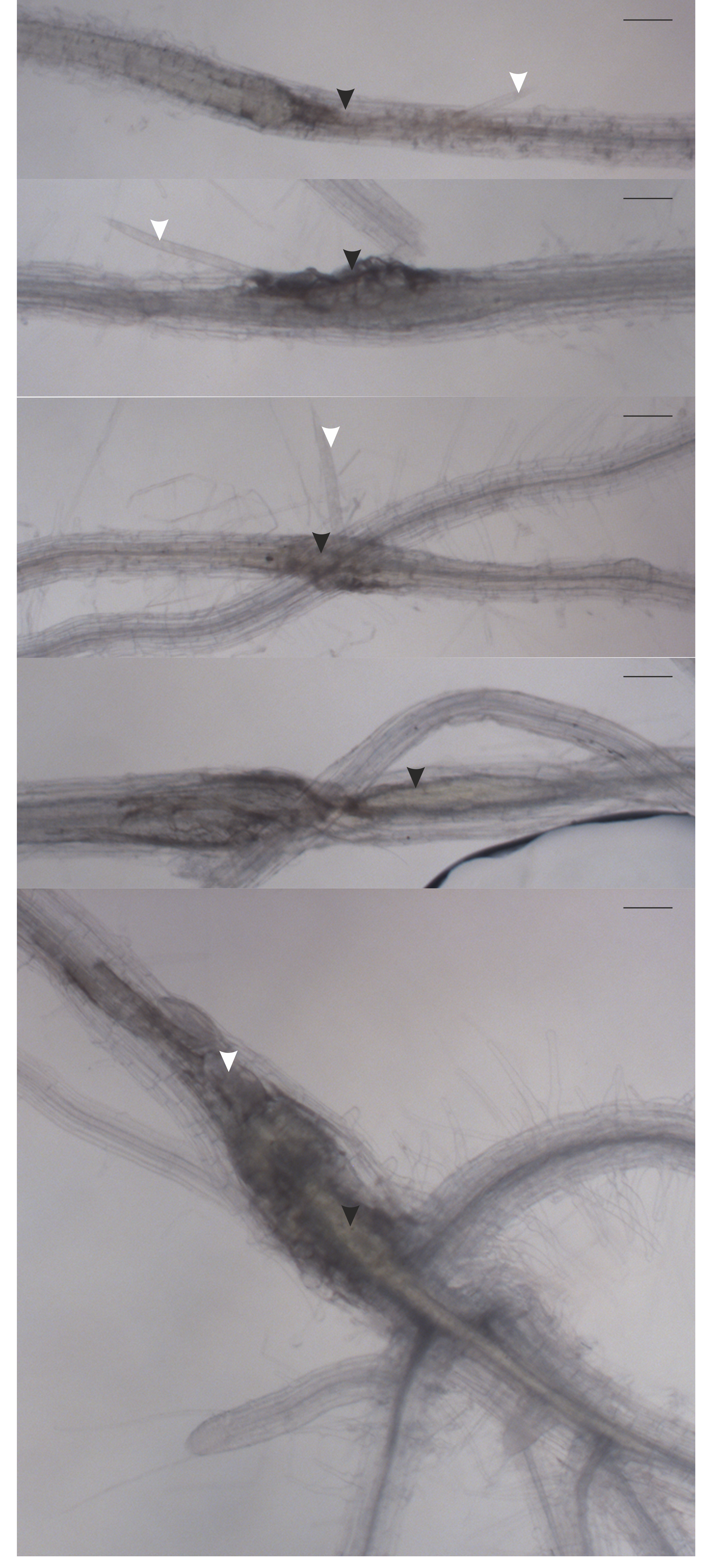

Supplement: S6 Fig — Nematodes (white arrows) and syncytia (black arrows) can be seen 10–12 days post infection displaying varying degrees of necrosis surrounding the syncytia and poor nematode development. It is not possible to measure the size of syncytia largely obscured by necrosis. (TIF) [file pgen.1007310.s006.tif]

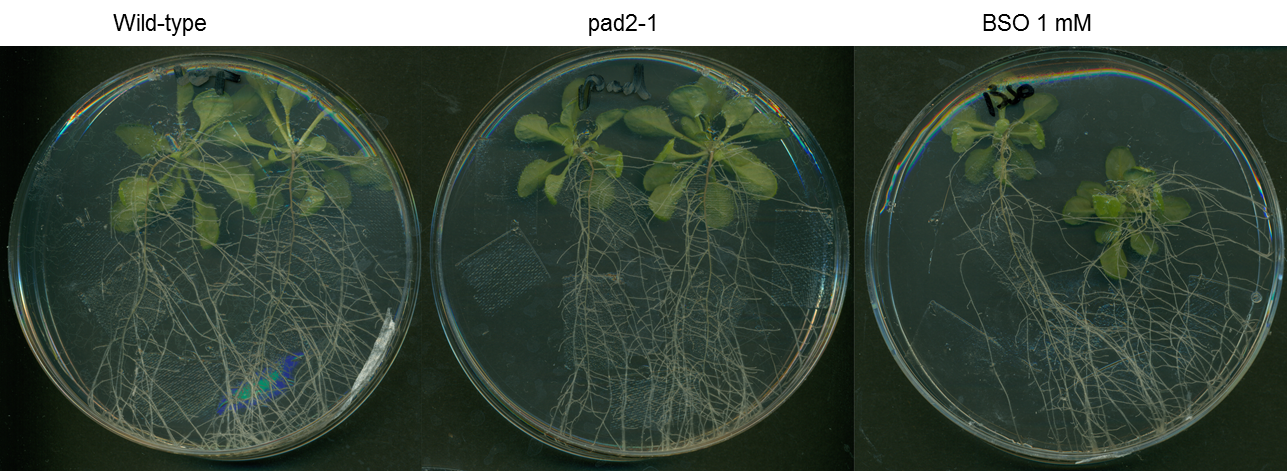

Supplement: S7 Fig — Root phenotype comparison between Arabidopsis wild-type, pad2-1 mutant, and wild-type plants treated with 1 mM BSO. (TIF) [file pgen.1007310.s007.tif]
